# Compact Gearboxes for Modern Robotics: A Review

**DOI:** 10.3389/frobt.2020.00103

**Published:** 2020-08-14

**Authors:** Pablo López García, Stein Crispel, Elias Saerens, Tom Verstraten, Dirk Lefeber

**Affiliations:** ^1^Robotics and Multibody Mechanics, Vrije Universiteit Brussel, Brussels, Belgium; ^2^Robotics and Multibody Mechanics, Flanders Make, Heverlee, Belgium

**Keywords:** transmissions, gearboxes, HRI, efficiency, virtual power, harmonic drive, cycloid drives

## Abstract

On the eve of Human-Robot-Interaction (HRI) becoming customary in our lives, the performance of HRI robotic devices remains strongly conditioned by their gearboxes. In most industrial robots, two relatively unconventional transmission technologies—Harmonic Drives© and Cycloid Drives—are usually found, which are not so broadly used in other industries. Understanding the origin of this singularity provides valuable insights in the search for suitable, future robotic transmission technologies. In this paper we propose an assessment framework strongly conditioned by HRI applications, and we use it to review the performance of conventional and emerging robotic gearbox technologies, for which the design criterion is strongly shifted toward aspects like weight and efficiency. The framework proposes to use virtual power as a suitable way to assess the inherent limitations of a gearbox technologies to achieve high efficiencies. This paper complements the existing research dealing with the complex interaction between gearbox technologies and the actuators, with a new gearbox-centered perspective particularly focused on HRI applications.

## Introduction

Industrial robots represent the backbone of several large-scale, traditional manufacturing industries including automotive or electronics. Today, many regions in the world see a realistic opportunity to bring back manufacturing industry introducing robots in Small and Medium size Enterprises (SMEs) and in assistive services, typically in healthcare (SPARC, 2015).

For large-scale, highly automated industrial environments, the advantage of robotic solutions compared to human operators mainly lies in (i) larger availability and (ii) the ability to move—typically large—payloads with extreme positioning accuracy and at high speed. These aspects are of pivotal importance when designing and selecting suitable technologies for an industrial robot, particularly for the prime movers and transmissions providing movement to these devices.

Applications in SME manufacturing and personal assistance challenge this traditional robotics paradigm. The key to success in these new applications lies in a very high degree of flexibility, required to enable a safe and efficient, direct cooperation with humans in order to achieve shared goals. This objective requires robots to first develop the ability to interact safely with humans, in a discipline usually referred to as pHRI—physical Human-Robot Interaction.

pHRI has a wide-ranging impact on robotic actuation. The experience accumulated during the past decades, mainly in healthcare robotics, indicates that for safe and efficient interaction with humans, robots need basically to move like humans, hence sacrificing some of their traditional advantages in terms of payload, accuracy, and speed. This situation has led to profuse research in the past years covering the optimal selection of prime movers and transmissions for HRI actuation (Zinn et al., 2004; Ham et al., 2009; Iqbal et al., 2011; Veale and Xie, 2016; Verstraten et al., 2016; Groothuis et al., 2018; Saerens et al., 2019).

Those works belong in a broader field of research studying the optimization of the coupling between prime mover and gearbox for a given task in automatic machines. A quick review of the main developments in this field provides useful insights to understand the impact of the gearbox on the overall system performance. Pasch and Seering (1983) identified the importance of inertia in actuation and proposed the use of a gear ratio to match the inertia of the motor and that of the reflected load as a means to minimize energy consumption for a purely inertial load. Chen and Tsai (1993) applied this idea to the field of robotics and identified the resulting acceleration capacity of the end-effector as a determining parameter. Van de Straete et al. (1998) separated motor and load characteristics to extend this approach to a general load and provided a method to identify suitable transmission ratios from a discrete set of motors and gearboxes. Roos et al. (2006) studied optimal actuator selection for electrical-vehicle powertrains adding the contribution of the gearbox efficiency. Giberti et al. (2010) confirm rotor inertia, transmission ratio, gearbox efficiency, and gearbox inertia as the most relevant parameters for actuation selection and propose a graphical method to optimize that selection for a dynamic task. Pettersson and Ölvander (2009) focused again on industrial robots and present a method which models the gearbox with a strong focus on mass, inertia, and friction. Rezazadeh and Hurst (2014) use a very accurate motor model and incorporate a fundamental bandwidth selection criterion, on addition to energy minimization. Dresscher et al. (2016) investigate the contribution of friction for a planetary gearbox in which Coulomb friction is the dominant friction mechanism and demonstrate how gearbox efficiency typically becomes dominant over motor efficiency at high transmission ratios.

From the initial gearbox models used in these works, where gearboxes are modelized as ideal transmission ratios, the complexity of the models increased progressively. Nevertheless, important—and unrealistic—simplifications need to be made to obtain good practicability in these methods. Important effects like those of torsional stiffness and lost motion are thus not incorporated, while gearbox inertia and efficiency models are strongly oversimplified. This is a justifiable approach for multiple applications, where simplified methods can help engineers select suitable transmissions. In HRI however, these properties are too pivotal for the suitability of the gearbox and they cannot be so strongly simplified.

A different approach is therefore required to provide useful guidance for gearbox selection in HRI, avoiding the excessive complexity of optimization tasks in this field. Providing detailed insight on the operational properties and performances of different gearbox technologies, to guide educated selection is another option, following the tradition of works like Schempf and Yoerger (1993) or Rosenbauer (1995). Following this approach, Siciliano et al. (2010), Li (2014), Scheinman et al. (2016), and Pham and Ahn (2018) provide interesting overviews on high precision gearboxes for modern robotics. However, the technologies are not analyzed in sufficient detail to gain a good understanding of the complex mechanisms in which they affect the performance of the robotic task.

The main objective of this review is consequently to complement these works with a detailed analysis of the underlying principles, strengths, and limitations of available technologies. Apart from enabling a forecast of the future of gearbox technologies in robotics, this approach can help gearbox non-specialists identify suitable compact gearbox technologies for the highly multi-factorial requirements of new robotic applications (López-García et al., 2018). For gearbox specialists from other domains, this analysis can help them gain useful insight in the particular needs of HRI applications.

This study begins with a brief description of the main requirements for future robotic transmissions, to introduce then an assessment framework designed to assess the suitability and potential of a particular gearbox technology for this field. This framework incorporates a strong pHRI perspective and incorporates a new parameter—Latent Power Ratio—to evaluate the inherent efficiency of a certain gearbox topology. This new framework is used in first instance to review traditional gearbox technologies used in industrial robots and of emerging transmission technologies which are currently in the process of finding their way into the market. Finally, a summary of the findings resulting from this review, together with our conclusions and recommendations, is given at the end of the paper.

## An HRI-Enhanced, Assessment Framework for Robotic Transmissions

### Control

The control of robotic devices is a very broad and complex topic, and the subject of extensive research literature. In this section we restrict ourselves to introducing the basic principles of Linearity and Reflected Inertia, which are basic to understand the gearbox influence on control.

Although in general speed and precision are conflicting requirements, conventional robotic devices excel in achieving high positioning accuracy at high speed thanks to the use of stiff actuators with very linear behaviors (Cetinkunt, 1991). The incorporation of a robotic transmission influences control complexity mainly in two ways: introducing additional non-linearities and strongly impacting the reflected inertias.

The non-linearities introduced by the incorporation of a transmission take basically the form of backlash and/or friction and reduce the system's bandwidth, creating important control challenges (Schempf, 1990). The statement *gears introduce backlash, friction, and (unwanted) compliance, which make accurate control difficult* (Hunter et al., 1991) is today just as valid as almost 30 years ago. For some technologies, large kinematic transmission errors and particularly non-linear friction behaviors can also induce considerable non-linearities.

Transmissions strongly impact a system's reflected inertias as well. In a robotic device, the inertia of the prime mover is typically several orders of magnitude smaller than that of the payload, a situation tending to make a system unstable and introducing strong control challenges. Adding a transmission strongly reduces the inertia of the payload seen by—reflected to—the prime mover by a factor equal to the squared reduction-ratio of the transmission. Thus, a careful selection of the transmission can result in more balanced inertias on both transmission's sides, contributing to minimize energy consumption and to more robust, stable, and precise system (Pasch and Seering, 1983).

Reflected inertias are particularly important when the end-effectors undergo rapid and frequent changes in speed and/or torque, a very common situation in automation and robotic tasks. In these cases, a bandwidth perspective is introduced to confirm the ability of the system to follow these changes (Sensinger, 2010; Rezazadeh and Hurst, 2014). This underlies the principle of backdrivability, the ability of a system to show low mechanical impedance when it is driven from its natural output (back-driven). This is particularly important in the frequent bidirectional energy exchange happening between a robot and its user, typical for rehabilitation devices or exoskeletons. As Wang and Kim (2015) demonstrate, a gearbox's backdrivability includes the combined effect of reflected inertia, reflected damping and Coulomb friction, and it is therefore strongly linked with the efficiency of the gearbox.

This highlights the importance in order to assess the control impact of a certain gearbox technology of both its transmission ratio capabilities and the non-linearities (backlash, friction) that it introduces.

### Safety

Industrial robots are traditionally placed behind fences, in highly structured environments where they can take advantage of their fast and accurate robotic movements without endangering the integrity of human operators.

A safe pHRI incorporating the ability to move safely in an unstructured/unknown environment is necessarily strongly linked to controllability. The current strategy used by roboticists to achieve this objective consists of *shaping the mechanical impedance* (Calanca et al., 2015), that is, letting a compliance-controller manage the complex dynamical relation between robot position/velocity and external forces (Hogan, 1984).

The principle is simple: to grant a good adaptation to an uncertain environment, as well as the integrity of the human operator/user during an interaction with a robotic device, the latter must move in a compliant, human-like manner (Karayiannidis et al., 2015). This underlines the importance of impedance and intrinsic compliance (De Santis et al., 2008) and explains the apparition of a new type of intrinsically flexible actuators for pHRI (Ham et al., 2009), where high compliance becomes desirable (Haddadin and Croft, 2016).

From a control perspective, the payload inertia reflected to the prime mover is reduced by a factor corresponding to the square of the gear ratio. In the same way, the typically small rotor inertia of the prime mover is amplified by this same factor when reflected to the payload side, which must be added to the inertia resulting from the movement of the robotic device and the load for safety considerations, further restricting the operating speeds.

Although most pHRI actuators today use high-ratio gearboxes, some reputed roboticists Seok et al. (2014), Sensinger et al. (2011) see a high potential for robotics in the use of high-torque (out-runner) motors requiring very small transmission ratios. New manufacturers of robotic solutions like Genesis Robotics from Canada, or Halodi Robotics AS from Norway, propose actuators for robotics based on these principles. According to them, increasing the motor's inertia and reducing the gear ratio should result in lower motor inertias reflected to the end-effector, thus enabling higher operational speeds and/or payloads without compromising the operator's integrity. Low ratios also have an additional bandwidth advantage: they have lower friction and backlash, reducing the non-linearities contribution from the gearbox. On the other hand, a moderate gear-ratio cannot compensate the non-linear coupling terms—typically cogging torque (Siciliano et al., 2010).

A closer look at the specifications of these new motors raises some questions in terms of attainable efficiency, weight or compactness, and on the hardware implications resulting from an extreme thirst for high electrical currents (HALODI Robotics, 2018; GENESIS Robotics, 2020).

Summarizing, there is no full agreement on how to best approach safe actuation for robotics. Yet, the strong natural ties between safety and controllability are as certain as the pivotal importance of the transmission's ratio and its non-linearities.

### Weight and Compactness

A lightweight design is of paramount importance to make safety and good performance compatible in the new robotics' applications (Albu-Schäffer et al., 2008). The latest Collaborative Robots (cobots) like KUKA's Lightweight-Robot, developed in collaboration with the Institute of Robotics and Mechatronics at the German Aerospace Center (DLR), live upon this principle and hence look very different to the heavy and bulky traditional industrial robots. Thanks to lower inertias, lightweight cobots enable higher productivities—higher speeds—without compromising user safety.

This advantageous aspect of a lightweight design has further advantages. For mobile robotic systems, lower weight means larger autonomies. In wearable, assistive robotic devices including prosthesis and exoskeletons, a lightweight design is also a key aspect to improve comfort (Toxiri et al., 2019).

High compactness is another characteristic shared by these new robotic devices: from cobots to assistive devices, being compact brings advantages in maneuverability and interaction comfort.

In robotic applications involving close cooperation with humans or the provision of mobile services, positions are inherently highly uncertain. Lightweight and compact designs are particularly advantageous (Loughlin et al., 2007) for these applications, with 2 fold consequences: prime movers and transmissions—typically the heaviest elements in a robotic device—need to be light and compact, but lightweight designs tend to demand lower torques.

In contrast to the weight of the gearbox, identifying a suitable criterion for assessing a gearbox's contribution to system compactness is more challenging. Physical volume definitely plays a role, but our experience demonstrates that the actual shape of the gearbox tends to have a larger impact. Another aspect worth mentioning here is the availability in some gearbox configurations of free space to allocate material or moving parts like electric motors or output bearings can also be of particular interest. We have therefore chosen to include in our evaluation framework the approximate shape (diameter × length) of the selected gearbox, while the availability of extra space can be directly assessed with help of the provided figures of each of the configurations.

### Efficiency and Virtual Power

#### Efficiency

In fields like automotive or wind turbines, gearbox efficiency has long been under strong focus. In robotics on the other hand, efficiency has not until very recently become a key decision parameter for the selection of a suitable gearbox (Arigoni et al., 2010; Dresscher et al., 2016).

Higher efficiencies—lower losses—enable lower energy consumptions and have a direct, positive contribution to both operation costs and to the environmental-footprint of a machine or device. For mobile and wearable robotic devices, better efficiencies help as well reduce the weight of the system—smaller batteries are required—and ultimately result in larger autonomies and better usability (Kashiri et al., 2018).

In gearboxes, there is one additional gain in going for lower losses: most mechanical transmissions used in robotics are form-closed and use some kind of teeth contact to transfer torque and movement between the prime mover and the end-effector. Owing to that, the kinematic ratio between input ω_In_ and output speeds ω_Out_ is locked by the number of teeth and defines its transmission ratio *i*_*K*_. In a gearbox with no losses, the torque ratio *i*_τ_ between output and input torques τ corresponds precisely to the inverse of kinematic transmission ratio, with opposed sign. But in a real gearbox, the presence of losses alters this equality, and because the kinematic transmission ratio is locked by the number of teeth, that the absolute value of the torque ratio must decrease proportionally with the losses:

$$\begin{array}{l} \frac{\omega_{In}}{\omega_{Out}}=\;i_{K}=-\;\eta \;i_{\tau }=-\eta \frac{\tau_{Out}}{\tau_{In}};where\;\eta \;represents\\ \text{ the system efficiency}. \end{array}$$

Consequently, high gearbox losses mean that less torque is available for the end-effector and larger transmission ratios are required to achieve the same torque amplification.

Gearboxes are subject to several types of losses. To classify them, we adopt the criteria proposed by Talbot and Kahraman (2014) and separate them into load-dependent (mechanical) power losses—originated by sliding and rolling of contact surfaces, both in the gear contacts and in the bearings—and load-independent (spin) power losses—originated through the interaction of rotating components with air, oil or a mixture of the two.

#### Virtual Power

The term Virtual Power was—to the best knowledge of the authors—originally coined by Chen and Angeles (2006), but this phenomenon explaining the anomalous high losses present in some planetary topologies has been known for long time under different names including *Blindleistung* (Wolf, 1958; Mueller, 1998) and *latent* or *futile power* (Macmillan and Davies, 1965; Yu and Beachley, 1985; Pennestri and Freudenstein, 1993; Del Castillo, 2002).

Owing to its operating principle, a gearbox always includes a high-speed, low-torque side and a high-torque, low-speed side. Its internal gear meshings are hence typically subject to either high-torque and low-speed or to high-speed and low-torque conditions. In some gearboxes though, owing to their specific topology, some gear meshings may encounter simultaneously high-speed and high-torque. Gear meshings can easily reach efficiencies above 98%, but because the generated losses are approximately proportional to the product of the relative speed of the two geared elements and the torque being transferred through the meshing (Niemann et al., 1975), unexpectedly large losses appear on those highly-loaded meshings. Virtual Power provides a framework to evaluate the contribution of this phenomenon, which we will hereafter refer to as the *Topological Efficiency* of a gearbox.

Several of the aforementioned authors propose methods to assess the topological efficiency of a given configuration and to derive its impact on overall system efficiency. In Chen and Angeles (2006) framework, *virtual power* is defined as the power measured in a moving—non-inertial—frame of reference. The *latent power* as introduced by Yu and Beachley (1985) corresponds accordingly to the virtual power when reference frame is the carrier element of the gearbox, while virtual power ratio is the ratio between the virtual power and the power generated by an external torque applied at a link. Using these elements, we define the *Latent Power Ratio* of a gearbox topology as the ratio between the sum of the latent powers in on all meshings, to the power input to the gearbox. A large latent power ratio therefore corresponds to low topological efficiency and indicates a strong tendency to generate large meshing losses.

In order to facilitate the understanding of the practical impact on overall efficiency of the topological efficiency—characterized by its Latent Power Ratio—of a given gearbox configuration, we use at this stage the equations proposed by Macmillan and Davies (1965) to calculate a simplified example.

A complete robotics' gearbox typically involves several meshing contacts, each with different operating conditions and parameters therefore resulting in different individual meshing efficiencies. These efficiencies are very high in optimized geared meshings—frequently above 99%—and allow us to simplify our calculations considering a generic, unique meshing efficiency of η_*m*_ = 99% in all the meshing contacts in our gearbox.

First, a reference gearbox, ideal in terms of topological efficiency, would have just one single meshing and a latent power ratio *L* = 1. The power losses inside this reference gearbox can therefore be easily calculated as a function of the input power as:

$$\begin{array}{l} P_{loss}=\;P_{IN}\;*\;\left(1-\eta_{m}\right) \end{array}$$

And the total meshing efficiency of the complete gearbox therefore corresponds to that of the single meshing contact:

$$\begin{array}{l} \eta_{sys,ideal}=\;\frac{P_{IN}-P_{Loss}}{P_{IN}}=\eta_{m}=99\%; \end{array}$$

A non-ideal gearbox with the same generic η_*m*_ in all its meshings, and with a Latent Power Ratio L characterizing its topological efficiency, indicates that the total losses in the gearbox can be approximated in first instance by:

$$\begin{array}{l} P_{loss,\;L}\approx \;P_{IN}*\text{ L }*\left(1-\eta_{m}\right)\; \end{array}$$

And the total meshing efficiency of the complete gearbox becomes now:

$$\begin{array}{l} \eta_{sys,L}=\;\frac{P_{IN}-P_{Loss,L}}{P_{IN}}\approx {\text{L }*\;\eta }_{m}+\left(1-L\right)\; \end{array}$$

Which for η_*m*_ = 99% and for a value of *L* = 50 results in:

$$\begin{array}{l} \eta_{sys,L}\approx \;50\% \end{array}$$

This result should be partially relativized because the accumulated losses in the first meshings engaged along the different internal power flows in a gearbox make that less virtual power as predicted by these equations will flow through the subsequent meshings. The effect of this is that the efficiencies will normally drop slightly less rapidly with Latent Power Ratio, and a more realistic value for the previous calculation would normally be between 55 and 60%.

To partially compensate this large impact of the topologic efficiency on the overall efficiency, configurations with large Latent Power Ratio therefore require extremely high meshing efficiencies: to achieve a system efficiency >70%, a system with *L* = 100 needs average meshing efficiencies above 99.5%.

In our further analysis we will therefore focus only in assessing the contribution of topological efficiency to the efficiency of a gearbox. This allows us to use a simplified method to calculate the latent power ratio which neglects in first instance the effect on the losses caused by the torque reduction. The corresponding calculations used to determine the latent power ratio of the different gearbox configurations analyzed in this work are included in Annex I.

Summarizing, in order to characterize the important effect of gearbox efficiency we will assess the order of magnitude of three parameters: (i) load-dependent losses, (ii) no-load starting torque, and (iii) latent power ratio. Although it is additionally affected by static friction and not only by Coulomb and viscous friction, we have selected the no-load starting torque (relative to the nominal torque) as a practical way to characterize load-independent losses. Our exchanges with gearbox manufacturers indicate that this is a common practice, it does not depend on the input power, and it is readily available in manufacturer's datasheet.

### Productivity

**Figure 1:**
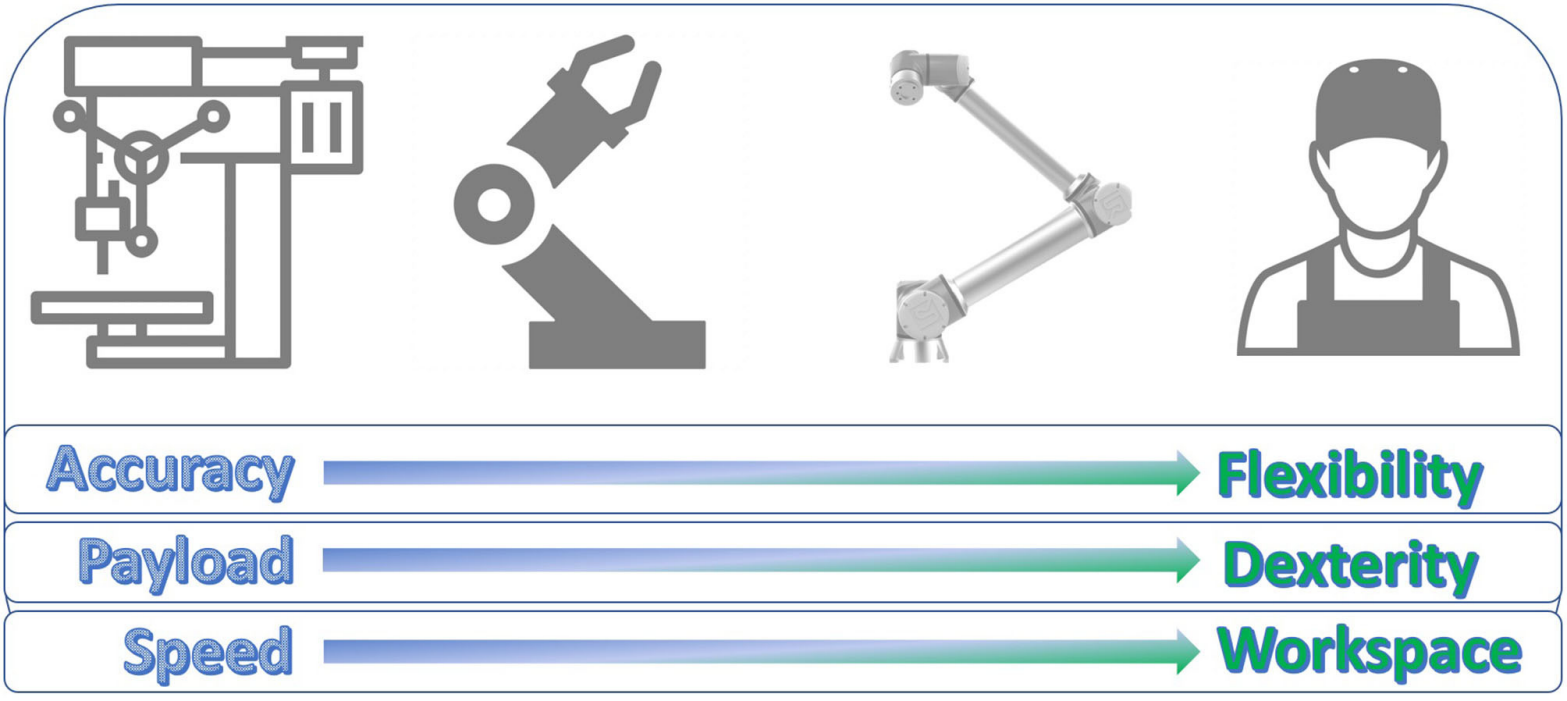
A graphical description of the transition of the main task objectives from machines through industrial robots and Cobots, up to human operators.

Compared to special-purpose and automatic-assembly machines, industrial robots cannot achieve the same standards of precision and speed. Both aspects had to be compromised to enable a larger degree of flexibility and mobility, and of the workspace (Rosenbauer, 1995). Seen from this perspective, HRI is just a further step in the same direction: in order to comply with further needs of flexibility and mobility in an unstructured environment, additional compromises are needed in terms of precision and speed. This transition is reflected in Figure 1.

#### Accuracy and Repeatability

Multiple aspects of a gearbox contribute to the resulting overall precision of a complete robotic device. These aspects have long been the focus of traditional robotics and are today well-understood, with works like those of Mayr (1989), Schempf and Yoerger (1993) or Rosenbauer (1995) providing very good references to understand these complex influences. Those studies identify the particularly important role played by lost motion and torsional rigidity.

Lost Motion is a further development of the principle of backlash which describes the total rotational displacement generated by the application of ±3% of the nominal input torque.

Torsional Rigidity characterizes the torsional compliance of all the elements in a gearbox involved along the complete force flow, under the influence of an external torque. It is established by means of blocking the gearbox input and progressively increasing the torque applied at the output, while changes in torsional stiffness—resulting in deviations from an ideally linear behavior—are registered.

Inherently precise—low lost motion and linear, high torsional rigidity—gearboxes simplify the control task and enable high precision ability, being ideally suited for position control, while less precise gearboxes put higher challenges to position control and can be used for more compliant actuation. In gearbox technologies where the speed has a strong influence on losses or with particularly non-linear friction behaviors, the contribution of this elements to accuracy must also be considered.

To characterize precision capabilities, our framework incorporates lost motion and torsional rigidity, together with a subjective assessment of the change in efficiency caused by speed/torque changes.

#### Speed and Payload

Industrial robots can handle large payloads at the cost of large inertias. For cobots on the other side, safety considerations imply that they are not expected to handle such large payloads, but thanks to lighter designs, they can actually achieve larger payload-to-weight ratios.

Safety considerations restrict also the extent to which this mass reduction can be exploited to increase the operational speeds (Haddadin et al., 2009). Yet, the lower torques promote the use of lighter and faster electrical motors, demanding in principle larger speed reduction ratios for these applications.

A criterion for characterizing a gearbox's contribution to speed and payload performance must reflect these aspects and motivates us to use in our framework (i) maximum input speed, (ii) maximum repeatable output torque—termed acceleration torque—and nominal torque, (iii) transmission ratio, and (iv) torque-to-weight ratios for both the nominal- and the acceleration torques.

### Summary

Characterizing robotic gearboxes is a challenging task: the high versatility of these devices, and their complex interactions with the prime movers and control systems, make a direct comparison of their performance particularly complex.

The transmission ratio has a demonstrated strong influence on the performance of a robotic system. This explains its preferent role in the literature dedicated to robotic actuation optimization, and the growing interest of roboticists in the possibilities to use variable transmissions (Kim et al., 2002; Carbone et al., 2004; Stramigioli et al., 2008; Girard and Asada, 2017). Although we are convinced that variable transmissions are very promising and will certainly contribute to shape the future robotics landscape, we have restricted our analysis here to constant-ratio compact gearboxes. At this point we believe that we are best served with this limited scope, which can actually contribute also to identify potential areas of applications and suitable technologies for variable-ratio transmissions.

Based on this analysis, we propose an assessment framework of future robotic gearboxes based on the following parameters:

- Transmission Ratio
- Acceleration- and nominal output torques
- Weight
- Shape: Diameter × Length
- Acceleration- and nominal torques-to-weight
- Efficiency: peak value and subjective dependency on speed and torque conditions
- Topological Efficiency: latent power ratio
- No-Load forward and backdriving starting torques in % of the nominal input torque
- Load-independent losses
- Lost Motion
- Maximal input speed
- Torsional rigidity

Our framework incorporates also a benchmark use case, representative for multiple pHRI tasks according to our own experience: acceleration torques above 100 Nm and gear ratios above 1:100, for which weight, compactness, and efficiency shall be optimized.

## Review of Transmission Technologies Currently Used in Industrial Robots

Electrical motors equipped with mechanical transmissions have typically been selected as actuators in robotics (Rosenbauer, 1995; Scheinman et al., 2016) also in industrial robots. These mechanical transmissions are almost inevitably based on some kind of gear technology (Sensinger, 2013).

Thanks to their larger ability to reduce the overall weight, and because electrical motors tend to have better efficiencies at high operating speeds, another characteristic of industrial robotic transmissions is the use of relatively large transmission gains (gear ratios), typically above 1:40 (Rosenbauer, 1995).

### Planetary Gearheads: an Extremely Versatile Platform

Planetary Gear Trains (PGTs) are compact, highly versatile devices broadly used in power trains. Due to their characteristic coaxial configuration and good power density, they are particularly suited for rotative prime movers like electrical motors.

**Figure 2:**
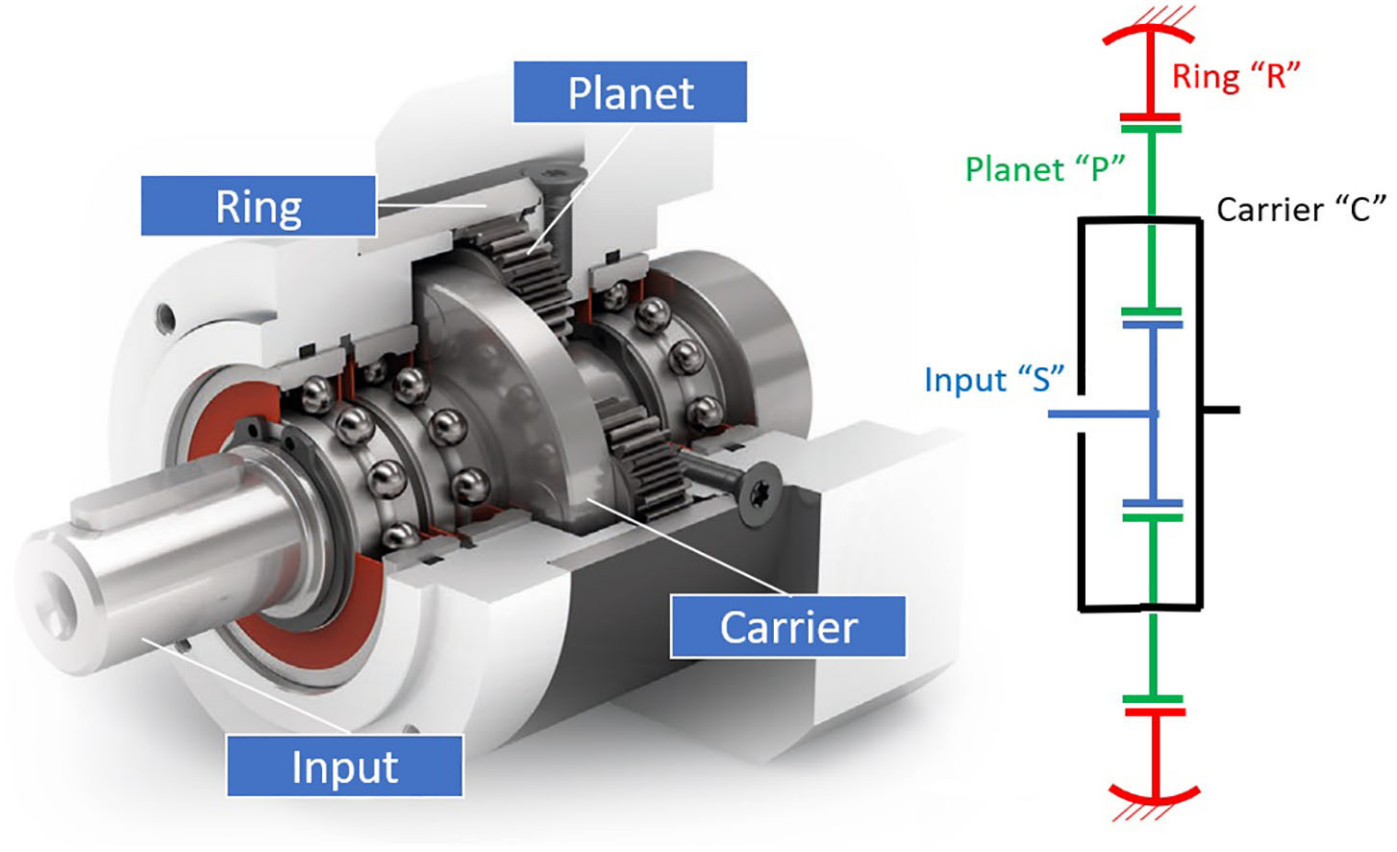
Internal arrangement of a Neugart gearhead indicating its main elements, adapted from Neugart (2020) with permission of © Neugart GmbH. It includes also a schema of its underlying topology.

PGTs can use two differentiated strategies to achieve high gains: (i) adding several stages of conventional, highly-efficiency PGTs—here termed gearheads and presented in Figure 2—or (ii) using particularly compact PGT configurations with the ability to produce high gear ratios.

While using several stages of gearheads makes best usage of the high gear meshing efficiencies and leads to highly efficient gearboxes, it typically results in heavy and bulky solutions. Compact PGT configurations on the other side can achieve high gear ratios in very compact shapes, but they suffer from surprisingly high losses derived from high virtual powers (Crispel et al., 2018).

**Figure 3:**
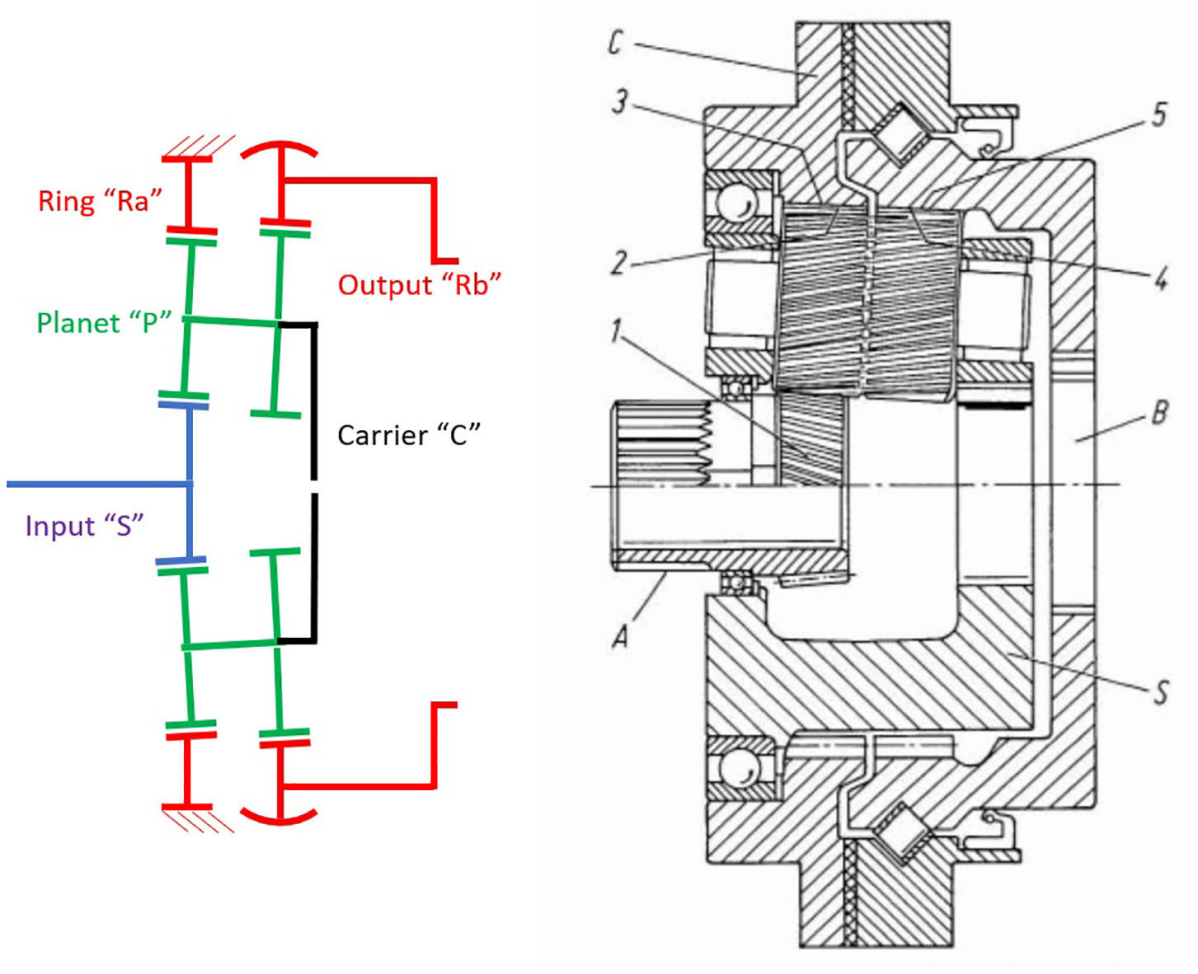
Internal arrangement of a ZF's RG Series Wolfrom PGT for robotic applications adapted from Looman (1996) with permission of © 1998 Springer-Verlag Berlin Heidelberg. It includes also a schema of its underlying topology.

A particularly compact PGT configuration for high ratios was first invented by Wolfrom (1912) and was used in the RE series gearboxes of the company ZF Friedrichshafen AG (ZF) aimed at industrial robotic applications (Looman, 1996). This configuration—shown on Figure 3—is strongly affected by Virtual Power and ZF's represents the only known commercial application of PGT configurations other than conventional gearheads. Although the manufacture of the RE series was discontinued in the 90's, Wolfrom PGT's are recently enjoying growing interest of the robotics research community, as we have summarized in a previous paper of the authors (López-García et al., 2019a).

Table 1 presents the PGT's assessment. Although over-dimensioned for our benchmark, we have used ZF's RG350 Wolfrom PGT to try to assess the potential of high-ratio PGT configurations, based on existing evidence of its suitability to achieve high-ratios (Arnaudov and Karaivanov, 2005; Mulzer, 2010; Kapelevich and AKGears LLC, 2013). For the gearheads we have selected—supported by the manufacturers—suitable solutions from the portfolios of Wittenstein and Neugart. Worth noting is the important role played by the maximum gear ratio per stage in a gearhead: while Wittenstein is closer to the feasibility maximum—given by contact avoidance between neighboring planets—Neugart selects in their PLE series (the PLFE series can reach 1:100 ratios in only two stages) a more restrictive approach and consequently needs three stages instead of two for Wittenstein, to achieve a total 1:100 gain. This leads to less compact solutions and lower efficiencies for a 1:100 application, but it allows Neugart to achieve higher gains—up to 1:512—without fundamental changes in weight, size, or efficiency.

**Table 1:** Assessment framework for planetary gear train solutions.

**PGTs**	**WITTENSTEIN (2020)—Alpha SP+075MF**	**Neugart (2020)—PLE 080**	**Looman (1996) ZF—RG350**
Transmission ratio	1:100 (2x stages)	1:100 (3x stages)	1:−76 (2x stages)
Acceleration/nominal torques	105/84 Nm	192/120 Nm	500^*^/350 Nm
Weight	3 kg	3.1 kg	6.4 kg
Shape	Φ95 × L120 mm	Φ80 × L168 mm	Φ160 × L90 mm
Torque-to-weight ratios	35/28 Nm/kg	62/39 Nm/kg	78/55 Nm/kg
Efficiency and subjective dependency on operating conditions	94%—low (speed and torque)	92%, low (speed and torque)	84%, low (speed and torque)
Latent power ratio (section/-s of Annex I including the calculations)	3.6 (GH, SGH)	4.7 (GH, SGH)	36.8 (WG)
No-load starting torque	0.5%^*^	0.7%^*^	1.5%^*^
Load-independent losses	5.5%	7.5%	14.5%
Lost motion	4–6 Arcmin	<11 Arcmin	()
Maximum input speed	8,500 rpm	7,000 rpm	5,000 rpm
Torsional rigidity	10 Nm/arcmin	8 Nm/arcmin	()

* *Values extrapolated and/or approximated, see further detail on Annex I*.

Gearheads show weights around 4 kg, which cannot be directly compared to the over-dimensioned RG350. The RG350 shows a shape with larger diameters and shorter lengths than the gearheads. In terms of torque-to-weight ratios, the values of both solutions appear to be relatively close.

Gearheads have a strong advantage in their good efficiencies (above 90%), which are less sensitive as well to changes in operating conditions, and the no-load starting torques are very low. High-ratio configurations show how a strong limitation in topological efficiency, resulting in lower efficiencies. This probably explains the why gearheads are today the dominant PGT- technology in robotics.

PGTs show the highest input speeds (up to 8,500 rpm), but their lost motion are also the largest (4–6 Arcmin) in conventional gearboxes. In robotics, PGTs were broadly used in the first industrial robots, while in the last decades their use has declined strongly mainly as a consequence of their limitations to reduce backlash. Although mechanisms exist to limit the inherently larger backlash of PGTs, those are practically based on the introduction of a certain pre-loading, negatively affecting their efficiencies (Schempf, 1990).

### Harmonic Drives: A Zero-Backlash, Lightweight Strain Wave Gearbox

The Strain Wave gearbox was invented by Musser (1955) and found broad application in the 70's, originally in aerospace. Its major space application was as the mechanical transmission element in the lunar rover vehicle on the Apollo 15, in 1971 (Schafer et al., 2005).

**Figure 4:**
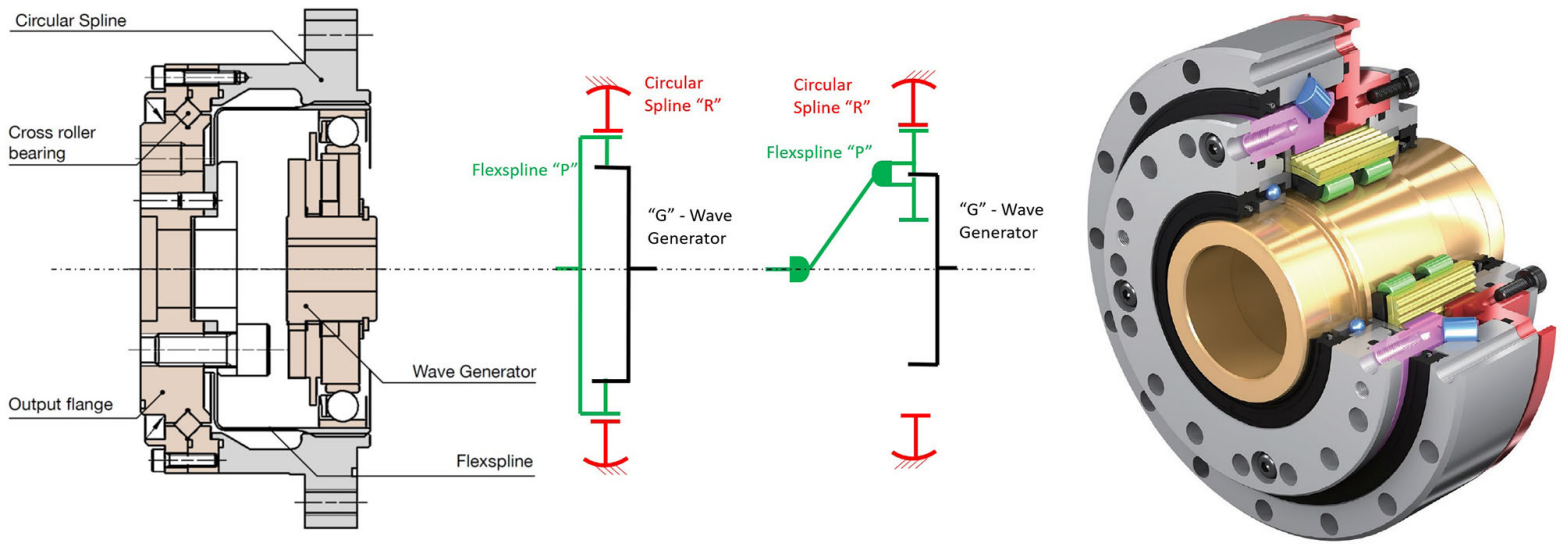
Internal configuration of a Harmonic Drive CSG gearbox (left), adapted from Harmonic Drive (2014) with permission of © 2019 Harmonic Drive SE, and a E-Cyclo gearbox (right) adapted from SUMITOMO (2020) with permission of © 2020 Sumitomo Drive Germany GmbH. The schema of their underlying KHV topology, used to develop its Latent Power Ratio calculations in Annex I, is also included.

Its name results from the characteristic deformation of its *Flexspline*, a non-rigid, thin cylindrical cup with teeth that serves as output. The Flexspline engages with a fixed solid circular ring with internal gear teeth, the *Circular Spline*, while it is deformed by a rotating elliptical plug—the *Wave Generator*, as it can observed in Figure 4. This type of gearbox is most commonly referred to as Harmonic Drive© (HD), owing to a very effective IP protection strategy.

For our benchmark analysis we have selected two suitable Harmonic Drive gearboxes, a CSD-25-2A meant for integration in a robotic joint to provide adequate structural boundary conditions, and an ultralight gear unit CSG-25-LW representing a structurally sufficient solution, which can be more directly compared to other technologies. Very recently SUMITOMO presented the new E-CYCLO gearbox, based as well of the strain wave principle of operation. SUMITOMO gave us access to its very recent catalog (SUMITOMO, 2020), enabling us to include it in our benchmark (Table 2). Another interesting Strain Wave, very similar to the Harmonic Drive, has recently been introduced as well by GAM to its robotics gearbox series, which includes as well planetary gear trains and cycloid drives (GAM, 2020).

**Table 2:** Assessment framework for strain wave solutions.

**Strain wave**	**Harmonic Drive (2014)—CSD-25-160-2A**	**Harmonic Drive (2014)—CSG-25-160-2UJ-LW**	**SUMITOMO (2020) E—CYCLO**
Transmission ratio	1:100	1:100	1:100
Acceleration/nominal torques	123/47 Nm	204/87 Nm	157/67 Nm
Weight	(0.24 kg)^a^	1.1 kg	1.6 kg
Shape	(Φ85 × L20 mm)^a^	Φ107 × L52 mm	Φ95 × L58 mm
Torque-to-weight ratios	(500/195 Nm/kg)^a^	208/79 Nm/kg	98/42 Nm/kg
Efficiency and subjective dependency on operating conditions	75%, high (speed and torque)	84%, high (speed and torque)	70%, high (speed and torque)
Latent power ratio	101 (SW)	101 (SW)	101 (PC)
No-load starting torque (forward and reverse direction)	17/20%	10/13%	45%/()
Load-independent losses	22% @ 500 rpm, nom. torque	18% @ 500 rpm, nom. torque	30% @ 500 rpm, nom. torque
Lost motion	<1 Arcmin	<1 Arcmin	<1 Arcmin
Maximum input speed	7,500 rpm	7,500 rpm	6,500 rpm
Torsional rigidity	9–17 Nm/arcmin	9–17 Nm/arcmin	11–16 Nm/arcmin

* *Values extrapolated and/or approximated, see further detail on Annex I*.

a *–this values refer to a unit not suitable as a standalone gearbox which requires additional structural support—directly impacting the identified characteristics—to be provided by the robotic device in which it is incorporated*.

The selected CSG model has a substantially larger torque capacity than targeted in our benchmark. The shape is characterized by larger diameters than lengths, while the weights are substantially lower than for other technologies and result in the best torque-to-weight ratios of the analyzed technologies. Indeed, the characteristic multiple tooth-engagement allows for larger torque resistance than in PGTs, making this technology a very good suit for the joints closer to the end-effector, where they are frequently found in today's industrial robots.

Peak efficiencies are lower than for gearheads and closer to the RG350, and efficiency is particularly sensitive to operating conditions. Strain Wave trains show large load-independent losses and no-load starting torques—particularly in back-driving conditions, which become particularly critical for high speeds and/or low torques (Harmonic Drive, 2014). For HRI robotic devices, subject to frequent speed and payload changes in combination with energy exchange between the robotic device and the user, this means that average efficiencies rapidly drop below 40–50% (López-García et al., 2019b). Worth noticing is also their large latent power ratio, indicating simultaneous presence of high torques and speeds in the teeth engagements, which helps also explain the relatively low efficiencies.

Thanks again to the multiple teeth engagement, lost motions below 1 arcmin can be reached and provide this gearbox with a strong advantage which helping Harmonic Drives find broad applications in industrial robots. They were able to displace PGTs from many applications, particularly after a major improvement of the performance resulting from a new teeth geometry introduced by this company in the 90's—which also improved its stiffness linearity (Slatter, 2000).

Maximal input speed used to be a strong limitation for the use of HD gearboxes in the past (Schempf, 1990), but new advances and design improvements allow them now to reach up to 7,500 rpm.

### Cycloid Drives: for High Robustness and Torsional Stiffness

**Figure 5:**
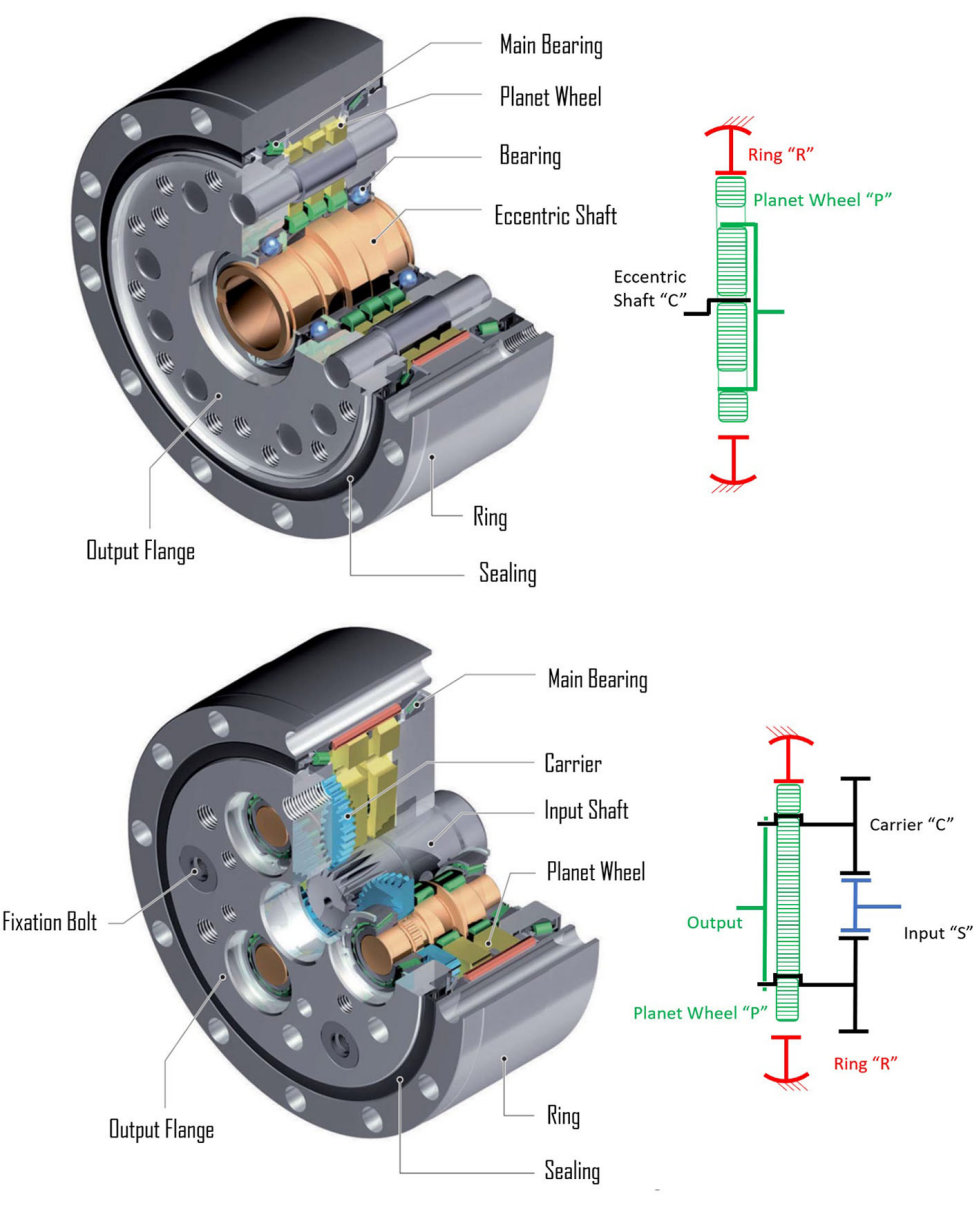
Internal configuration of a SUMITOMO Fine Cyclo F2C-A15 and a Fine Cyclo F2C-T155 cycloid drives identifying its main elements, adapted from SUMITOMO (2017) with permission of © 2017 Sumitomo Cyclo Drive Germany GmbH. It includes also a schema of its underlying topologies.

Since their invention by Lorenz Braren in 1927 (Li, 2014), cycloid drives have found application mainly in boats, cranes, and some large equipment as steel strip rolling trains or CNC machines. In cycloid drives, an eccentric input motion creates a wobbly cycloidal motion of a single, large planet wheel, which is then converted back in a rotation of the output shaft and results in a high reduction capacity (Gorla et al., 2008), see Figure 5.

Table 3 includes the market leader (NABTESCO RV) in this segment and the main challengers (SPINEA and SUMITOMO). The RV from NABTESCO and the Fine-Cyclo T-series of SUMITOMO include a pre-gearing, conventional PGT stage. The payload capability of these devices is larger than required for our benchmark and results in large weights. This provides already a valuable insight: more compact solutions are not available in the market and are—according to the information provided by some of the manufacturers—less interesting because they would need extreme manufacturing precision and ultimately result in high costs.

**Table 3:** Assessment framework for cycloid drive solutions.

**CYCLOID drives**	**NABTESCO (2018)—RV-25N**	**SPINEA (2017)—TwinSpin TS110**	**SUMITOMO (2017)—Fine CYCLO F2C-T155**	**SUMITOMO (2017) Fine CYCLO F2C-A15**
Transmission ratio	1:108	1:119	1:118	1:89
Acceleration/nominal torques	612/245 Nm	244/122 Nm	417/167 Nm	335/111 Nm
Weight	3.8 kg	3.8 kg	4.8 kg	2.7 kg
Shape	Φ133 × L62 mm	Φ110 × L62 mm	Φ126 × L68 mm	Φ126 × L60 mm
Torque-to-weight ratios	161/64 Nm/kg	64/32 Nm/kg	87/29 Nm/kg	124/41 Nm/kg
Efficiency and subjective dependency on operating conditions	87%, high (speed and torque)	74%, high (speed and torque)	87%, high (speed), medium (torque)	87%, high (speed and torque)
Latent power ratio	33.8^*^ (CG)	120 (PC)	29.2^*^ (CG)	90 (PG)
No-load starting torque	16% (@ 500 rpm)	19/27%	23% (@ 500 rpm)	64/67%
Load-independent losses	13%	25%	13%	13%
Lost motion	1 Arcmin	<1 Arcmin	<0.75 Arcmin	<1 Arcmin
Maximum input speed	()	4,500 rpm	8,500 rpm	5,600 rpm
Torsional rigidity	61 Nm/arcmin	>22 Nm/arcmin	25–41 Nm/arcmin	15–28 Nm/arcmin

* *Values extrapolated and/or approximated, see further detail on Annex I*.

Shapes are similar to those of strain wave gearboxes, while weights are larger and closer to those of the PGTs, for the aforementioned reasons. Torque-to-weight ratios are larger than those of PGTs but slightly lower than for strain wave gearboxes. The main advantage of cycloid drives lies precisely in their ability to withstand large loads and particularly impact loads, and in the little maintenance required.

Peak efficiencies are larger than for strain wave gearboxes and closer to those of PGTs, but efficiency is highly dependent on operating conditions (Mihailidis et al., 2014) and both the no-load start torques and the latent power ratio are high, both similar to strain wave gearboxes.

Although they tend to present some backlash, such if often compensated for in their design to reach levels comparable to those of the strain wave gearboxes, probably at the cost of slightly higher frictions. Their torsional rigidity is the largest of the analyzed gearbox technologies.

Cycloid drives have an inherent limitation to cope with high input speeds, caused by the presence of a large and relatively heavy planet (cam) wheel resulting in large inertias and imbalances. This motivates the use of typically two planet wheels, arranged in series and shifted 180 degrees to each other, to cancel out imbalance, reduce vibrations and enable larger input speeds. This explains how, by means of combining cycloid drives with pre-gearing stages consisting of conventional PGTs stages enabled cycloid drives to achieve their current broad acceptance in robotics. This arrangement improves efficiency, reduces sensitivity to high input speeds and provides for easy adaption of their gear ratios. In the 90's Harmonic Drives dominated the robotic gearbox market, but the improvements in cycloid technology enabled cycloid drives to start gaining terrain, first in Japan and then elsewhere (Rosenbauer, 1995). Nowadays manufacturers like NABTESCO, SUMITOMO or NIDEC propose cycloid hybrids integrating a PGT pre-gearing cover over 60% of the robotic gearbox market, and have therefore become the new dominant technology, particularly for proximal joints subject to higher loads and lower weight restrictions (WinterGreen Research, 2018).

Finally, the presence of a relatively large torque-ripple which introducing non-linearities and complicating their control is also worth mentioning. This torque ripple is linked to the necessity of using cycloid tooth profiles to avoid teeth interference between the large planet wheel/-s and the ring gearwheel, making these devices extremely sensitive to the center-distance variations produced by even small manufacturing errors. Several attempts to improve this situation exist, using involute teeth—less sensitive to center-distance variations—with reduced pressure angles and/or contact ratios to minimize radial forces and improve efficiency (Morozumi, 1970), as well as using other forms of non-involute teeth (Koriakov-Savoysky et al., 1996; Hlebanja and Kulovec, 2015).

## Review of Emerging Transmission Technologies for Robotics

### The REFLEX Torque Amplifier

Genesis Robotics has drawn a lot of attention in the robotics community with the arrival of their direct-drive motor, the *LiveDrive*^©^. According to Genesis, the LiveDrive in the two available topologies—radial and axial fluxes—provides benchmarking performance in Torque-to-Weight ratio. The axial flux motor can achieve up to 15 Nm/kg, while the radial flux up is limited to maximum 10 Nm/kg.

**Figure 6:**
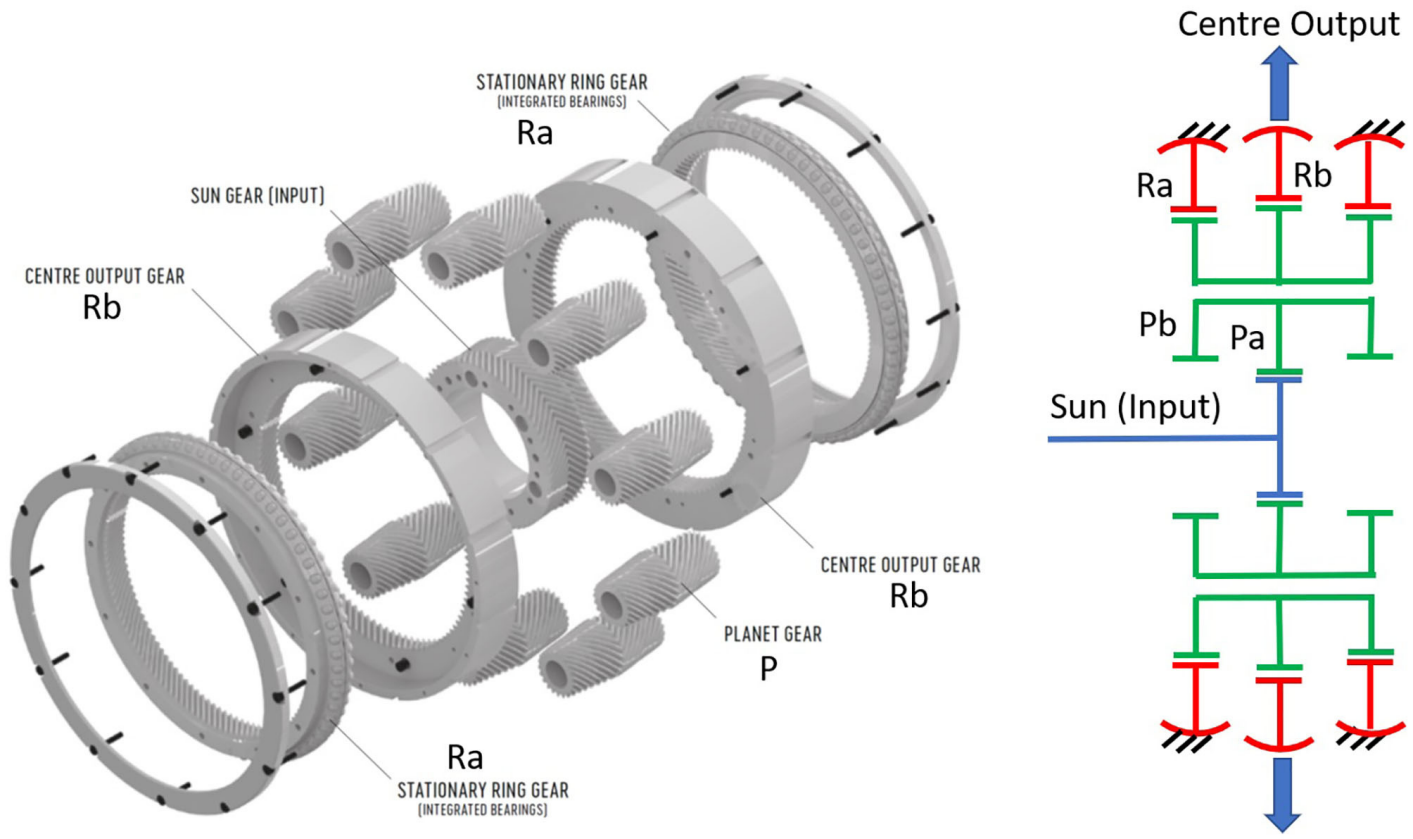
Internal configuration and main elements of a Reflex gearbox adapted from GENESIS Robotics (2020) with permission of © 2019 Genesis Robotics. It includes also a schema of its underlying topology.

To enlarge its application spectrum, Genesis Robotics introduced a compatible gearbox termed *Reflex*, which is shown in Figure 6. This injection-molded, ultralight plastic gearbox is targeted at lightweight robots and although it was initially designed to work together with the LiveDrive and is therefore targeted at gear ratios below 1:30, it is also capable of providing larger gear ratios up to 1:400 (GENESIS, 2018).

The underlying topology is that of Wolfrom PGT with multiple, smaller planets (Klassen, 2019), in which the reaction (stationary) ring gearwheel is split into two for balancing purposes, following a design originally proposed by Rossman (1934) and used as well in the Hi-Red gear of Tomcyk (2000).

In the Reflex gearbox, the output ring is also split to facilitate the assembly with helical teeth. Another interesting aspect of this design is the taped shape of the planets, which the authors suspect to be linked to the possibility of preloading the system in order to achieve the zero–backlash that Genesis claims is possible with this gearbox. The flexibility of the plastic planet wheels also provides an advantage for the reduction of the backlash, according to the company.

Unfortunately, independent tests are not available yet to confirm the given performances and no official data particularly on efficiency is for now available from Genesis, which is why Table 4 includes only the Latent Power Ratio value resulting from its topology.

**Table 4:** Assessment framework for emerging gearbox technologies.

**Emerging technologies**	**GENESIS—reflex torque amplifier**	**IMSystems—archimedes drive**	**FUJILAB—bilateral drive**
Achievable transmission ratios	1:30 (up to 1:400)	1:100 (up to 1:500)	1:96 ()
Acceleration/nominal torques	87/44 Nm	125/100 Nm	120/() Nm
Weight	0.76 kg	1.1 kg (embedded solution)	1.3 kg
Shape	Φ160 × L54 mm	Φ1500 × L80 mm	Φ94 × L62 mm
Torque-to-weight ratios	115/58 Nm/kg	113/91 Nm/kg	92/() Nm/kg
Efficiency and subjective dependency on operating conditions	()	()	90%, low (torque and speed)
Latent power ratio	22 (80 for 1:100) (WG)	80 (WG)	21 (WG)
No-load starting torque (forward and reverse direction)	()	()	<0.1%
Load-independent losses	()	()	1%^*^

* *Values extrapolated and/or approximated, see further detail on Annex I*.

In summary, although the underlying Wolfrom topology indicates that efficiency will certainly be a complex challenge to solve, this innovative gearbox illustrates the large potential available for rethinking existing technologies and adapting those to the future needs in robotics. Genesis Robotics has recently entered an interesting partnership with established industrial companies as Koch Industries Inc. and Demaurex AG.

### The Archimedes Drive

IMSystems from the Netherlands is a spin-off of the Delft University of Technology, created in 2016 to exploit the invention of the *Archimedes Drive* (Schorsch, 2014).

**Figure 7:**
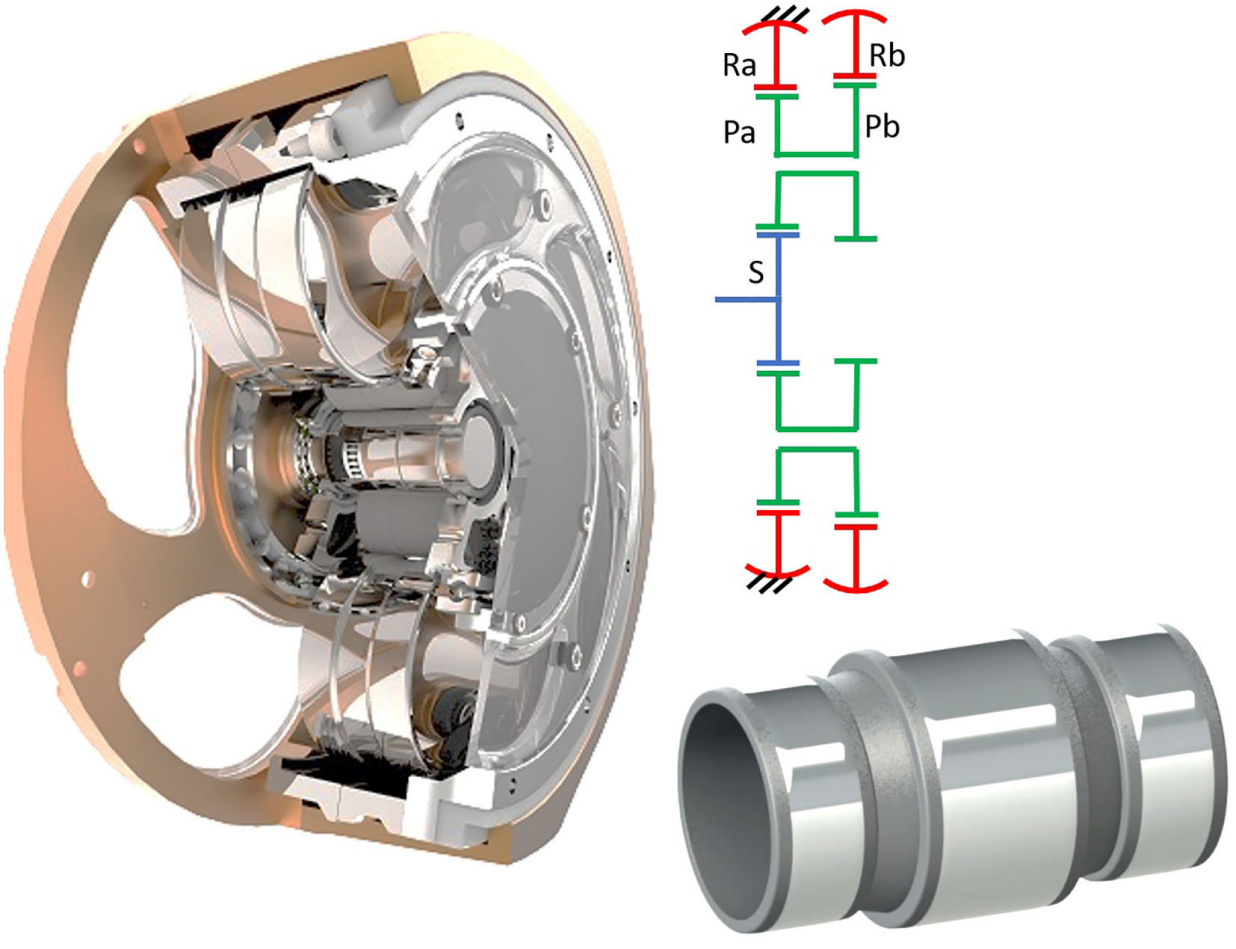
Internal configuration of the Archimedes Drive with a detail showing its Flexroller planets adapted from IMSystems (2019) with permission of © 2019 Innovative Mechatronic Systems B.V., with a schema of its underlying topology.

The Archimedes Drive follows again the topology of a Wolfrom gearbox (also with a split reaction ring gear in some of its designs) but incorporates a breakthrough innovation in the use of rollers instead of gearwheels, to replace teeth contacts with rolling contacts, see Figure 7. The controlled deformation of the roller-planets enables the transmission of the torque between the planets, in a similar way as the wheels of a vehicle.

The performance shown in Table 4, extracted from the company's brochure (IMSystems, 2019) and available on demand, shows that the use of a Wolfrom topology provides this device with the ability to reach very high gear ratios in a compact shape, but it also results in low topological efficiency. According to IMSystems, the replacement of gear-teeth contact with rolling contact contributes to the minimization of the contact losses, which particularly in the torque transfer between the planet and the ring rollers should compensate for the high Latent Power Ratio, and result in maximum efficiencies around 80% (IMSystems, 2019). No data is provided in terms of starting torques or load-independent losses.

To enable a high torque transfer without slip, the deformation of the planet rollers as well as the manufacturing tolerances of the gearbox must be tightly controlled. This represents one of the main technological challenges, and it is the core of the innovation introduced by this technology (Schorsch, 2014).

### The NuGear

STAM s.r.l. is a private engineering company based in Genova which helped develop a robotic joint for the I-Cub humanoid robot. Their NuGear is a nutating gearbox which was originally conceived (Barbagelata and Corsini, 2000) targeting space applications, but could develop its potential for robotics as well through the exploration of alternative manufacturing means.

No information is yet publicly available about the performance characteristics of this gearbox, which means that we can only provide here a preliminary analysis of its topology and the resulting performances which can be expected based upon the limited information available basically from the Caxman EU project (CAxMan, 2020) for which the NuGear was a use case, and from the available patents (Barbagelata et al., 2016).

**Figure 8:**
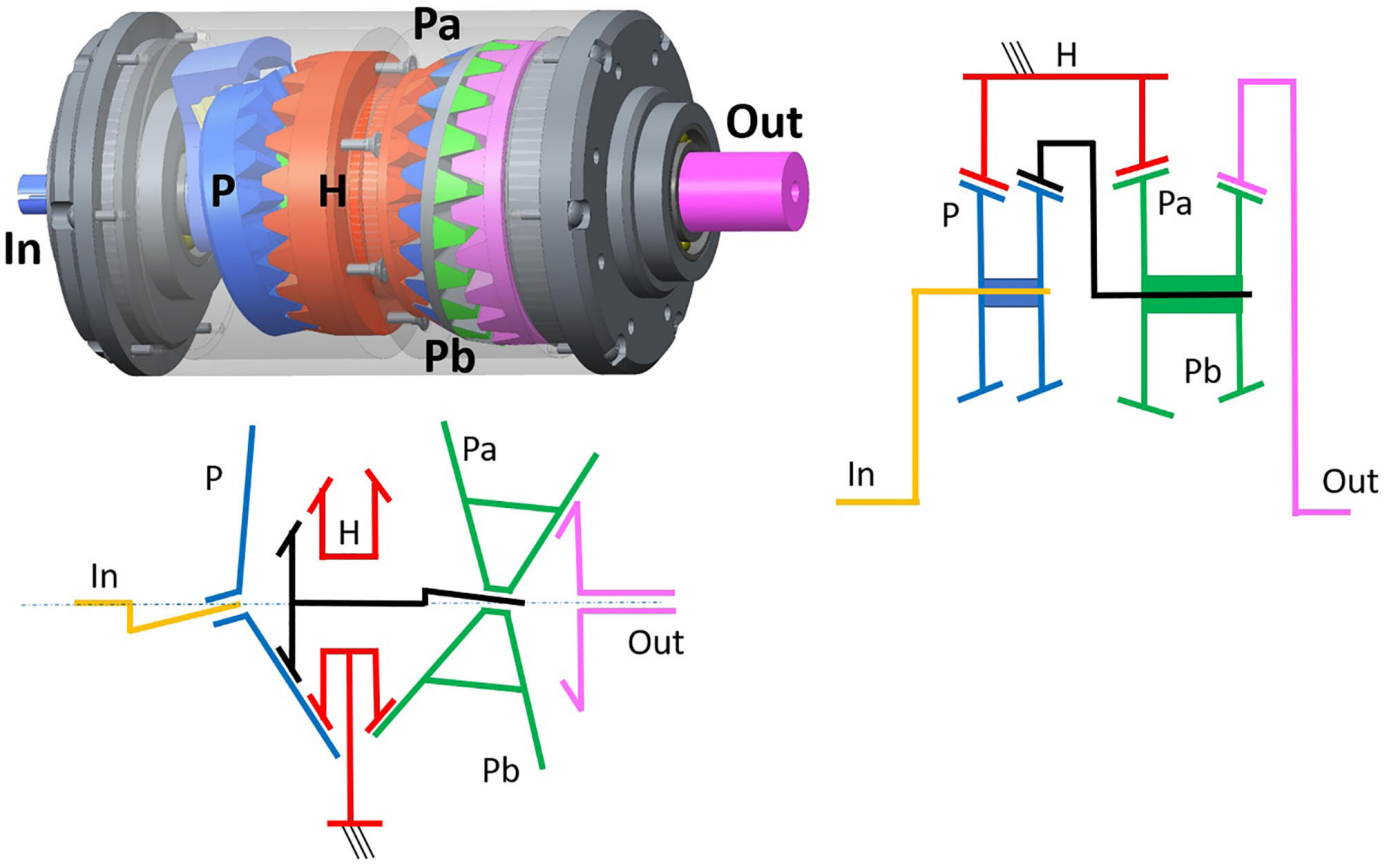
Internal configuration of a two-stage NuGear gearbox for the version with opposed planet contacts adapted from CAxMan (2020) with permission of © Stam S.r.l. It includes also a schema of its underlying topology.

In Figure 8 the internal structure of the NuGear is presented using an equivalent PGT configuration—abstracting the nutating aspect to ease the understanding. By doing so it becomes clear that a NuGear resembles two Wolfrom PGTs for which the carrier is used as the input, connected in series and where each of them corresponds to one of the two stages defined in Barbagelata et al. (2016). This indicates again that a relatively high Latent Power Ratios will be present in this gearbox. For a gear ratio of 1:100 and assuming a balanced gain of 1:10 on each of the two stages, as proposed in Barbagelata et al. (2016), we obtain using the equations derived in Annex I a latent power ratio of 32 indicating similar topological efficiency to that of a Wolfrom PGT.

It remains to be confirmed to which extent the use of Additive Manufacturing methods can help STAM s.r.l. reduce the large manufacturing cost of the bevel gears, and whether the nutating operation can achieve sufficient reliability and a more compact shape, which could open the door to its usage in the field of robotics (CAxMan, 2020).

### The Bilateral Drive

The FUJILAB in Yokohama proposed in Fujimoto (2015) a highly backdrivable gearbox for robotics, which would be particularly suited for operation without need for a torque sensor (Kanai and Fujimoto, 2018).

**Figure 9:**
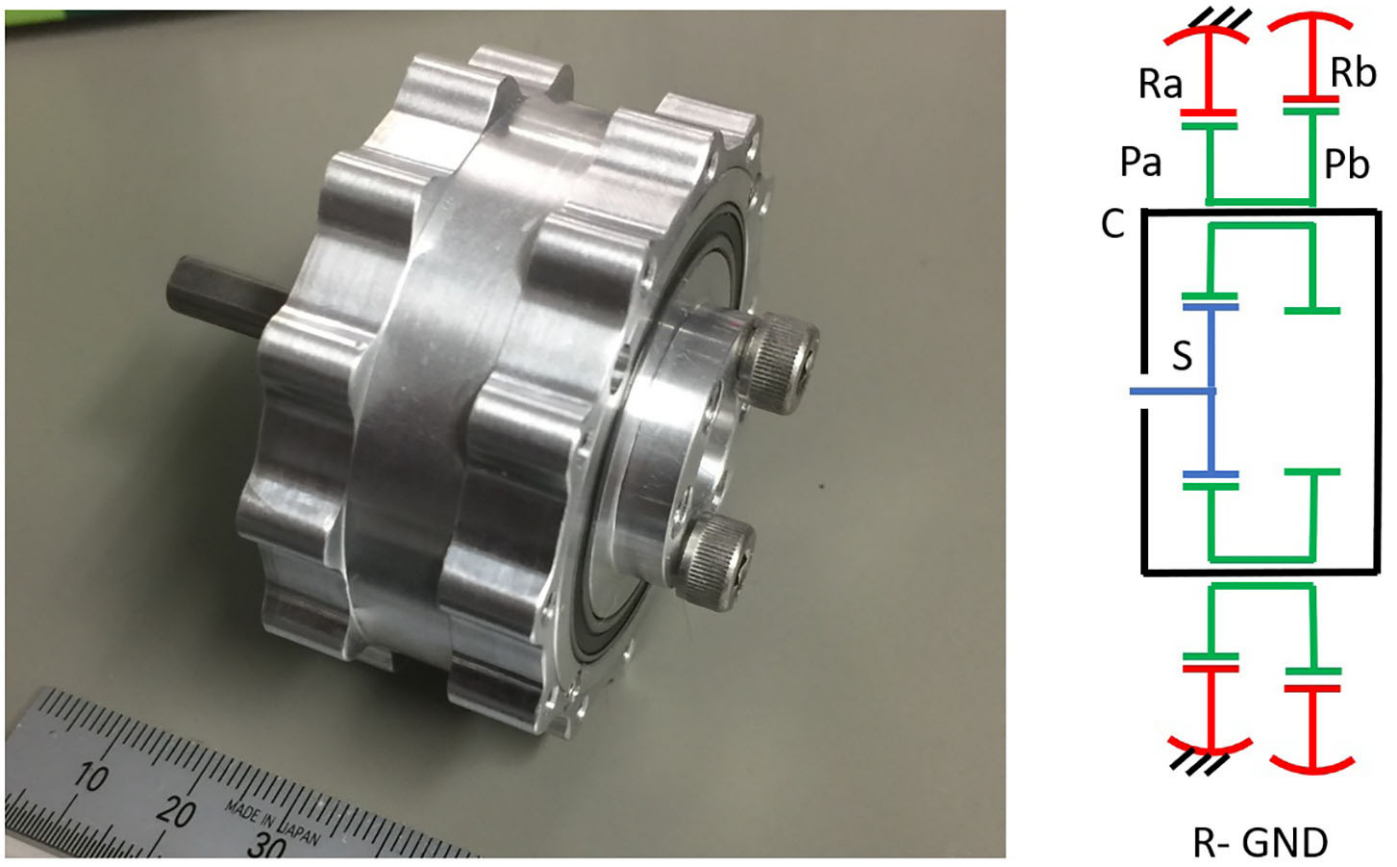
Internal configuration of a Bilateral Drive, a highly efficient gearbox capable of achieving 1:102 gear ratios using a Wolfrom topology, courtesy of © Yasutaka Fujimoto.

As it can be observed in Figure 9, the configuration of this device is again that of a Wolfrom PGT. With this topology, Fujimoto et al. were able to reach, for a 1:102 gear ratio, forward efficiencies of 89.9% and backdriving efficiencies of 89.2%. The No-Load Starting torque in backdriving direction amounted to 0.016 Nm in a gearbox with an outer diameter of ~ Φ50 mm (Kanai and Fujimoto, 2018). The strategy followed to reach such high efficiencies with a Wolfrom topology consists on the optimization of the profile-shift coefficients (Fujimoto and Kobuse, 2017).

These promising results—see Table 4—indicate that equalizing the approach and recess ratios through optimization of the profile-shift coefficients can lead to extremely high meshing efficiencies. To the best of the authors' knowledge, this strategy was originally proposed by Hori and Hayashi (1994) and is particularly interesting in a Wolfrom topology, where it could ultimately enable efficiencies above 90% in combination with high-gear ratios and compact topologies.

### The Gear Bearing Drive

Following the pioneering work in this field of John M. Vranish from NASA, which resulted in the invention of a carrier-less planetary gear in Vranish (1995) and of the partial tooth gear bearings (Vranish, 2006), the NASA Goddard Space Flight Center presented its concept of a new Gear Bearing Drive in Weinberg et al. (2008).

**Figure 10:**
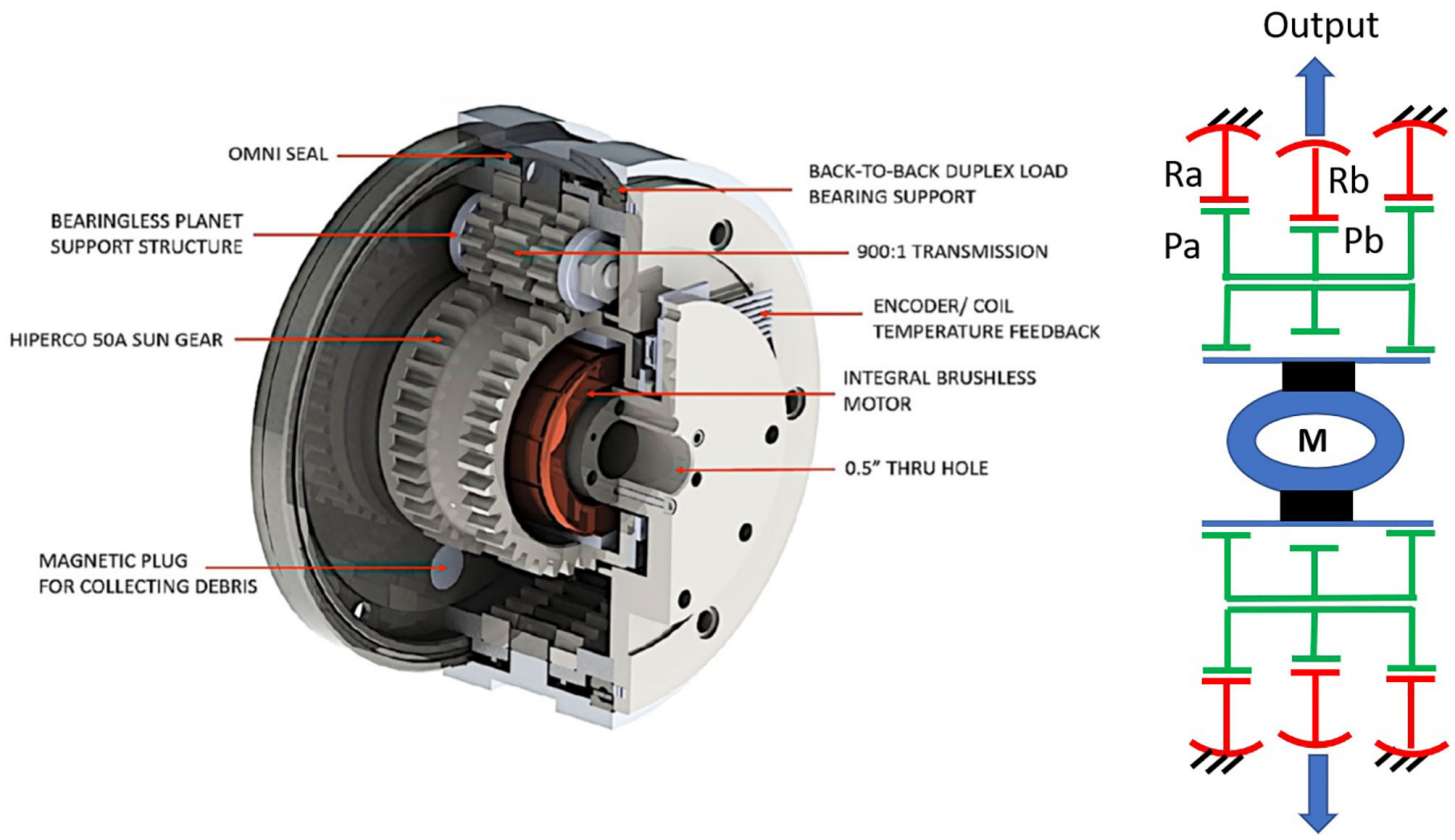
Internal configuration of the Gear Bearing Drive, including the embedded brushless motor adapted from Brassitos and Jalili (2017) with permission of © 2017 American Society of Mechanical Engineers ASME. On the right the underlying Wolfrom topology with a split reaction ring is also shown.

The Northeastern University in Boston continued the development of this new actuator for applications in robotic joints. As it can be observed in Figure 10, it incorporates a Wolfrom gearbox adapted to include Vranish's carrier-less design and gear bearings. The gear bearings are rolling contacts which are provided for each pair of meshings gears corresponding to their pitch diameter and reduce the load on the gearbox bearings (Brassitos et al., 2013). This topology enables a convenient integration of an electromotor, which is therefore embedded in the hollow area provided inside a large sun gearwheel in a configuration particularly aimed at space applications (Brassitos and Jalili, 2017).

In Brassitos and Jalili (2018) a metal prototype of a Gear Bearing Drive with a gear ratio of 1:40 is characterized in terms of stiffness, friction and kinematic error. The measurements are very in line with those of the FUJILAB and confirm the low no-load starting torque of this configuration (0.0165 Nm for an outer gearbox diameter of ~Φ100 mm). After experimentally measuring the stiffness, friction and kinematic error of their drive, (Brassitos and Jalili, 2018) integrated those values into a dynamic model which was then simulated and compared to the open loop velocity response of the system under free sinusoidal motion, showing good correlation, and suggesting a very convenient high linearity in the transmission.

Preliminary measurements indicated good combined efficiencies for the motor and the Wolfrom gearbox with a gear ratio of 1:264 (Brassitos et al., 2013), which do not correlate very well with a calculated Latent Power Ratio of 196. Efficiency has not been again in the focus of the recent papers of the authors and we have unfortunately not been able at this point to confirm the final efficiency levels that the newer prototypes can reach.

In any case, the Gear Bearing drive brings in very interesting propositions to exploit the potential of the Wolfrom topology in robotics. The possibility to eliminate the carrier and embed an electric motor inside the gearbox, in a shared housing, results in impressively compact designs. The possibility of using gear bearing pitch-rollers to reduce radial loading on the bearings is as well a promising option for improving compactness, and to increase efficiency (Brassitos et al., 2019).

### The Galaxie Drive

Schreiber and Schmidt (2015) protects the main innovations included in the Galaxie Drive, a gearbox which WITTENSTEIN is currently bringing into the precision gearbox market through its start-up Wittenstein Galaxie GmbH, created in April 2020.

**Figure 11:**
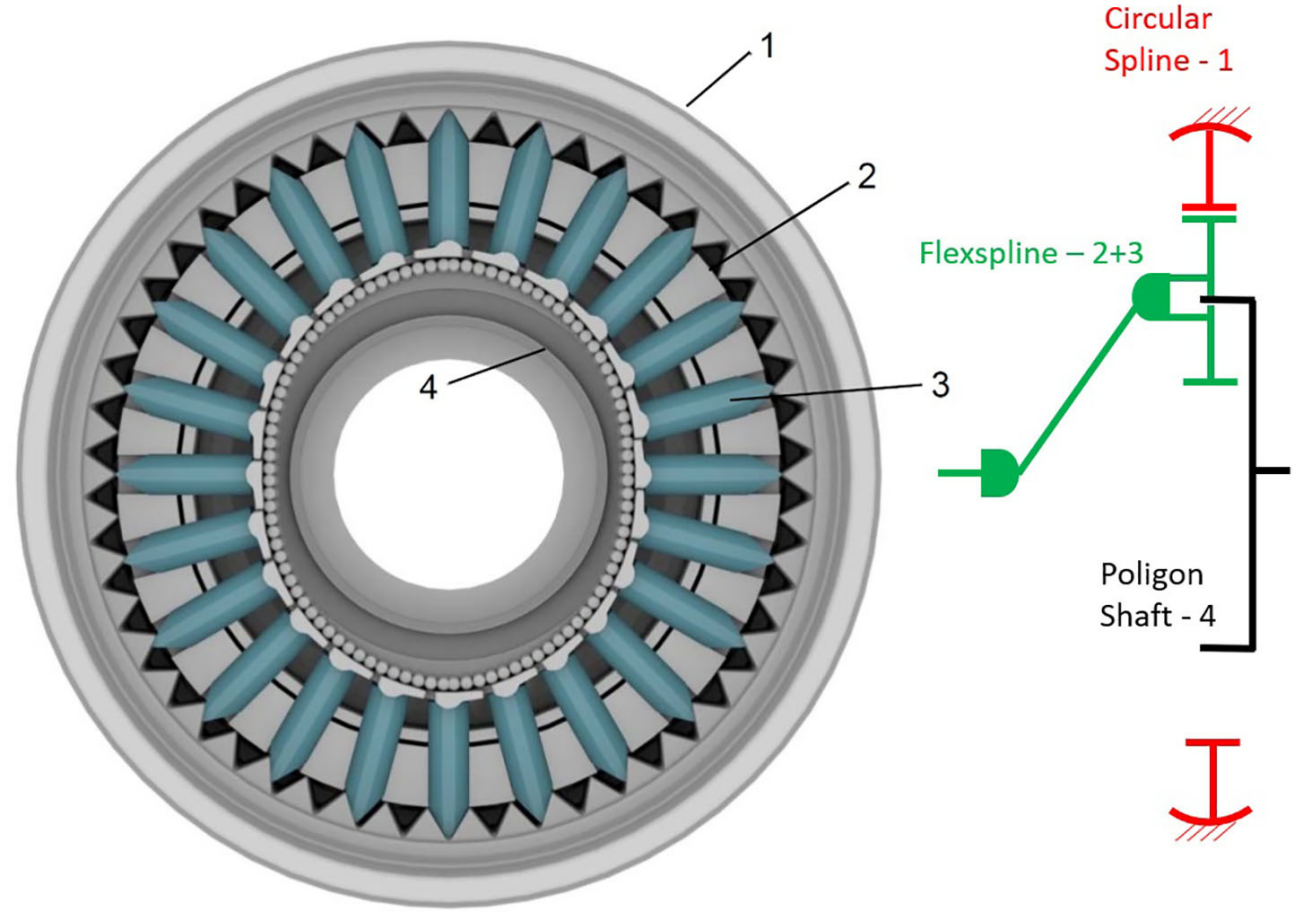
Detail of the teeth engagement of a Galaxy (R) DF gearbox adapted from Schreiber (2015) with permission of © 2020 Wittenstein Galaxie GmbH. It includes a schema of the underlying KHV topology.

Although datasheet and detailed information are not yet available, the principle of operation and expected gains have also been disclosed. The Galaxie Drive introduces a new kinematic approach based on a linear guidance of the singular tooth in a *Teeth Carrier*, but according to these authors its topology resembles that of a Strain Wave Gear, see Figure 11. The flexspline is replaced by a Teeth Carrier including two rows of individual teeth, arranged to move radially and engage with the circular spline as a rotating *Poligon Shaft* makes the role of a wave generator with polygonal perimeter (Schreiber and Röthlingshöfer, 2017). Multiple, individual teeth are consequently engaged simultaneously with the circular spline—just as in a Harmonic Drive. This, together with the highly torque-resistant two-point contact between each single tooth and the Teeth Carrier, provide this device with a characteristic zero-backlash, high torsional stiffness and a benchmark torque-to-weight ability, according to the manufacturer.

In a direct exchange, Wittenstein's representatives confirmed that the apparent issue of friction between the individual teeth and their guiding Circular Ring is solved and the Galaxie can reach peak efficiencies above 90%. Owed to its underlying KHV configuration, large Latent Power Ratios are expected, but it is not possible yet to gain further insights on the meshing efficiency that will result from the radial movement of the teeth, which incorporates a new logarithmic spiral tooth flank (Michel, 2015).

Originally the Galaxie Drive is targeted at precision machinery, where the high rigidity and torque resistance can help increase the speed and improve the productivity. In the future, we will certainly be able to assess the potential of this innovative technology as well for robotic applications.

## Discussion

A new generation of robotic devices is changing priorities in the selection of adequate gearboxes. Instead of extreme precision at high speeds, these devices impose stronger requirements in terms of lightweight and very efficient mechanical gain devices.

The ultralight strain wave drives (HD, E-cyclo) are certainly in a very good position to serve these needs, a fact confirmed by its current dominance in the field of cobots. When considering a strain wave drive for a pHRI robotic task, operation at low torques and speeds shall be reduced to a minimum if efficiency is to be maximized. Although their optimized teeth geometry contributes to a more linear torsional stiffness, friction remains highly non-linear and direction-dependent, inducing as well certain usage limitations. Ratcheting as a consequence of impact loading is a further limitation to consider for this type of gearbox, which the E-Cyclo should not present (SUMITOMO, 2020).

Cycloid Drives have come a long way to ultimately become the dominant technology in industrial robots. Through technological advances to improve their backlash and input speed limitations, they can now provide good accuracy with acceptable efficiency—despite of high Latent Power Ratios, resulting from an underlying KHV topology equivalent to that of the strain wave drives. The use of a pre-gearing stage provides an important contribution as well to this objective by means of improving underlying topological efficiency. Ultralight designs like that of SPINEA show interesting potential, but eventually more disruptive approaches like plastic materials will be required to suit the needs of lighter gearboxes and larger gear ratios needed for HRI. Until this is possible, Cycloid Drives can only be considered for large payloads, where their larger weight and resulting inertias are not critical to function. When extreme accuracy is not needed, backlash compensation measures can be avoided in favor of better efficiencies and lower start-up torques. Care shall in any case be taken to adequately manage torque ripple, and the pre-gearing stage will probably need to stay in order to enable high input motor speeds.

The impossibility of Planetary Gearboxes to reduce backlash maintaining good performance and limitations in torsional stiffness has limited their use in industrial robotics. Yet, PGT's are extremely versatile, as their extensive usage in multiple modern industrial devices demonstrates. And they are inherently efficient, reliable, and relatively easy—cheap—to manufacture. This may explain the recent interest of roboticists in PGTs, and why five of the six highly innovative gearboxes studied here are based on a high-ratio, PGT configuration: the Wolfrom topology. A better topological efficiency combined with improvements on meshing efficiency with profile modifications or going even one step further to replace teeth with rolling contacts are promising features. In combination with the possibilities opened up by their hollow topology, these elements could potentially drive a PGT come-back in robotics.

Our research indicates that the large versatility of the gearbox technologies involved in robotics represent a major challenge for a direct comparison of their performances. As the examples of backlash and maximum input speed show, adequate design modifications can suitably compensate most of the original weak points of a certain technology, at the cost of making compromises in other aspects typically including efficiency, size, weight, and cost. In the same way, large Latent Power Ratios indicate a significant topological disadvantage in terms of efficiency, but such can also be—at least partially—compensated for with adequate modifications. A learning effect of this is therefore that the selection of a suitable gearbox technology for a certain pHRI application is an extremely complex process demanding for a deep understanding of the fundamental weaknesses, improvement potentials, and derived compromises of each technology. Our initial research objective to contribute with a simple selection table capable of guiding unexperienced robotic engineers in the selection of suitable gearbox technologies for their robotic devices could consequently not be achieved. Instead, this paper collects and explains the main selection parameters and their related challenges in each of the available technologies, aiming at helping pHRI robotic engineers to develop the required skills necessary for an educated choice of a suitable, individually-optimized gearbox.

Two important aspects of robotic gearboxes for pHRI could unfortunately not be adequately assessed in our research at this stage: noise and cost. As robotic devices get closer to humans, noise is receiving more and more attention from roboticists. Gearboxes certainly represent an important source of noise (airborne and structure borne), but unfortunately two main limitations recommended to exclude noise from our analysis at this stage. First, most gearbox manufacturers do not provide yet quantitative noise performance evaluations and when they do, those tend to follow different testing methods which are also not particularly suited for the operating conditions in pHRI. Second, current gearbox technologies still have to undergo a pending noise optimisation process.

Cost is as well an important parameter to make pHRI technologies more available and becomes therefore essential for the selection of suitable gearboxes for future robotic technologies. Unfortunately, here again insufficient background information is available to the scientific community in order to enable a systematic a fair assessment of the large-scale cost potential of a certain gearbox technology. Before a suitable framework to assess this potential can be defined, a large amount of research work is required which clearly exceeded the scope of our investigation.

These two limitations outline the main recommendations of the authors for interesting future lines of research. Defining standardized testing conditions for airborne and structure borne noise in gearboxes, particularly adapted to typical operating conditions and need in pHRI, could enable a direct comparison of different technologies and contribute to their noise optimization. Additionally, compiling available cost models for the manufacturing processes involved in the manufacture of gearboxes, and adapting those to the specificities of the particular technologies used in robotics, would enable putting together a framework to evaluate the large-scale cost potential (and barriers) of the different technologies.

## Author Contributions

All authors have been involved in the preliminary work related to this research topic and contributed to the conceptualization of the framework presented in the manuscript. PG worked on the derivation of a suitable assessment framework to perform the gearbox analysis and took the lead in writing the manuscript and shaping it into its current form. PG and ES contributed equally to identify potentially suitable technologies, and on their analysis with the aid of the framework. All authors proof read and contributed to the final version of the paper.

## Conflict of Interest

The authors declare that the research was conducted in the absence of any commercial or financial relationships that could be construed as a potential conflict of interest.
