# Supplementary material for: Compact Gearboxes for Modern Robotics: A Review
Source: Front Robot AI. 2020 Aug 14;7:103. doi: 10.3389/frobt.2020.00103 (PMC7806062; doi:10.3389/frobt.2020.00103)
Supplement: Supplementary file 1 [file Table_1.DOCX]

Supplementary Material

# Notations

$\omega_{j}=rotational speed of a certain element \text{j}\text{ }ofa gearbox$

${\omega^{'}}_{j}=rotational speed of element \text{j}, seen from a inertial reference frame (typically the$

$carrier of the gearbox)$

$i_{G}=gear ratio of a gearbox configuration G= \frac{\omega_{input element k}}{\omega_{output element j}};$

$P_{j}=input power to a certain gearbox element j$

$\tau_{j}=input torque to a certain gearbox element j$

$L_{G}=latent power ratio of a certain gearbox configuration G$

*R = Ring gearwheel*

*S = Sun (pinion) gearwheel*

*P = Planet gearwheel*

*GH = Conventional Gearhead*

*SGH = Gearbox composed on multiple stages of Conventional Gearheads*

*WG = Wolfrom Gearbox*

*SW = Strain Wave gearbox*

*PC = Pure Cycloid gearbox*

*CG = Cycloid Gearbox with pre-gearing*

# Latent Power Ratio Calculations

## Conventional Gearhead (GH), one stage

Carrier “C”

Input “S”

Planet “P”

Ring “R”

To study the kinematics, we separate the PGT movement into a “rolling movement” – seen from a non-inertial reference frame fixed on the carrier – and a “coupled movement”:

- Coupled: ${\omega"}_{c}=\omega_{c}= {\omega"}_{s}={\omega"}_{R};$
- Rolling: ${\omega'}_{c}=0$; ${\omega'}_{R}=\omega_{R}-{\omega"}_{R}=-\omega_{c}=\frac{1}{i_{GH}}\omega_{s};$

Assuming no losses to simplify the calculation, power balance implies:

$\sum P_{j}=0=\tau_{S}\omega_{s}+\tau_{C}\omega_{C};$ $\tau_{C}=-\tau_{S}\frac{\omega_{s}}{\omega_{C}}=-\tau_{S}i_{GH};$

Where $i_{GH}$ corresponds to the gear ratio of the gearhead, and $\tau_{j}$ the external torque applied to the gearhead element “j”.

Torque Balance implies, assuming again no losses:

$\sum\tau_{j}=0=\tau_{S}+\tau_{C}+\tau_{R}=>\tau_{R}=-\tau_{S}-\tau_{C}={\left( i_{GH}-1 \right)\tau}_{S};$

And the Latent Powers P’ in the meshings S-P and R-P can be calculated as:

$${P'}_{S-P}=\tau_{S}{\omega^{'}}_{s}=-\tau_{S}\omega_{s}\left( 1-\frac{1}{i_{GH}} \right)=P_{S}\left( 1-\frac{1}{i_{GH}} \right);$$

$${P'}_{R-P}=\tau_{R}{\omega^{'}}_{R}=\tau_{S}\omega_{s}\left( i_{GH}-1 \right)\left( \frac{1}{i_{GH}} \right)=P_{S} \left( 1-\frac{1}{i_{GH}} \right);$$

And the Latent Power Ratio expressed as the sum of the absolute values of the Latent Powers on all meshings, divided by the Input Power results:

$$L_{GH}=\frac{\sum\left| {P'}_{j} \right|}{P_{S}}=2\left( 1-\frac{1}{i_{GH}} \right);$$

## Conventional Gearhead, multiple stages (SGH)

$$P_{S}$$

$$P_{C}$$

$$i_{S1}$$

$$i_{S2}$$

$$i_{S3}$$

…

Considering again no losses, we can use the previously derived equation for each of the stages and calculate the total Latent Power Ratio of the gearhead as:

$$L_{SGH}=\sum L_{Sj}=2\sum\left( 1-\frac{1}{i_{SJ}} \right);$$

##
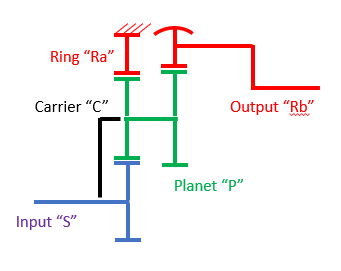
Wolfrom Gearbox (WG)

In a Wolfrom PGT, the shaft “S” is used as the input while a first ring gearwheel “Ra” is fixed and a second ring gearwheel “Rb” becomes the output of the system. Using the same “rolling movement”- framework analogue as for the gearhead, we obtain:

$$\sum P_{j}=0=\tau_{S}\omega_{s}+\tau_{Rb}\omega_{Rb};$$

$$\tau_{Rb}=-\tau_{S}\frac{\omega_{s}}{\omega_{Rb}}=-\tau_{S}i_{WG};$$

${\sum\tau_{j}=0; \tau}_{Ra}=-\tau_{S}-\tau_{Rb}={\left( i_{WG}-1 \right)\tau}_{S};$

$$\omega_{c}=-{\omega'}_{Ra}=\frac{Z_{s}}{{(Z}_{Ra}+Z_{s})}\omega_{s};$$

And the Latent Powers in the meshings S-P, Ra-P and Rb-P can be calculated as:

$${P'}_{S-P}=\tau_{S}{\omega^{'}}_{s}=-\tau_{S}\omega_{s}\left( \frac{Z_{Ra}}{Z_{Ra}+Z_{s}} \right)=P_{S} \left( \frac{{-Z}_{Ra}}{Z_{Ra}+Z_{s}} \right);$$

$${P'}_{Rb-P}=\tau_{Rb}{\omega^{'}}_{Rb}=\tau_{S}\omega_{s}\left( {-i}_{WG} \right)\left( \frac{1}{i_{WG}}-\frac{Z_{s}}{{(Z}_{Ra}+Z_{s})} \right)=P_{S} \left( i_{WG}\frac{Z_{s}}{Z_{Ra}+Z_{s}}-1 \right);$$

$${P'}_{Ra-P}=\tau_{Ra}{\omega^{'}}_{Ra}=\tau_{S}\omega_{s}\left( 1-i_{WG} \right)\left( \frac{Z_{s}}{{(Z}_{Ra}+Z_{s})} \right)=P_{S} \left( 1-i_{WG} \right)\left( \frac{Z_{s}}{{(Z}_{Ra}+Z_{s})} \right);$$

Assuming $i_{WG}$< 0 as it is the case for ZF’s RG350 gearbox, the Latent Power Ratio expressed as the sum of the absolute values of the Latent Powers on all meshings, divided by the Input Power results:

$$L_{WG}=\frac{\sum\left| {P'}_{j} \right|}{P_{S}}=1+\frac{Z_{Ra}+Z_{S}\left( 1-2i_{WG} \right)}{Z_{Ra}+Z_{S}};=2\left( 1-i_{WG}\frac{Z_{S}}{Z_{Ra}+Z_{S}} \right);$$

Or, if we alternatively use the Carrier “C” as the input of the system (NuGear Drive):

$${P'}_{C,Rb-P}=\tau_{Rb}{\omega^{'}}_{Rb}=\tau_{C}\omega_{C}\left( {-i}_{WG} \right)\left( \frac{1}{i_{WG}}-1 \right)=P_{C} \left( i_{C,WG}-1 \right);$$

$${P'}_{C,Ra-P}=\tau_{Ra}{\omega^{'}}_{Ra}=\tau_{C}\omega_{C}\left( -1+i_{WG} \right)\left( -1 \right)=P_{C} \left( 1-i_{C,WG} \right);$$

And the Latent Power Ratio:

$$L_{C,WG}=\frac{\sum\left| {P'}_{j} \right|}{P_{C}}=2\left| 1-i_{C,WG} \right|;$$

##
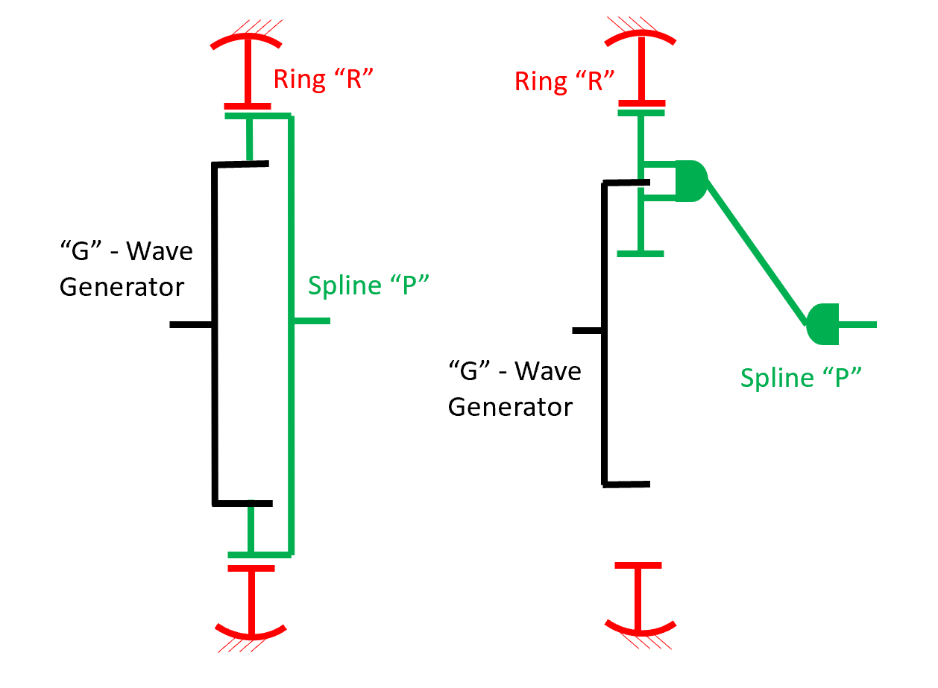
Strain Wave (SW) Gearbox

A Strain Wave Gearbox can structurally be assimilated to a Planetary Gearhead for which the Wave generator is the carrier and the deformable Spline is the only planet. The Wave Generator is the input, and the Spline is the output of the gearbox. This type of configuration is usually termed KHV.

The Latent Power in the meshing between the Ring and the Spline can be calculated as:

$${P'}_{R-P}=\tau_{P}{\omega^{'}}_{P}=\tau_{Out}\left( \omega_{Out}-\omega_{In} \right)=P_{S} \left( 1-i_{SW} \right);$$

Where $i_{SW}$ corresponds to the gear ratio of the Strain Wave and is a negative value for this type of planetary gearbox configuration. The Latent Power Ratio becomes:

$$L_{SW}=\left| \left( 1-i_{SW} \right) \right|;$$

## “Pure” Cycloid (PC) Gearbox


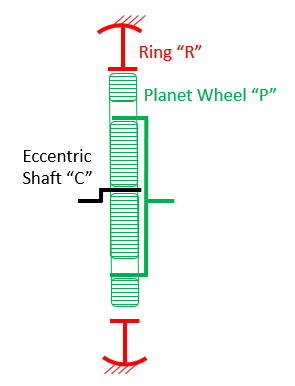
A pure cycloid gearbox can be assimilated as well to an KHV planetary gearbox, where the input eccentric shaft plays de role of the carrier, and second eccentric shaft is used to extract the rotation of the planet as the output…

This means that the equations of the Wave Strain remain valid and the Latent Power Ratio becomes:

$$L_{PC}=\left| \left( 1-i_{PC} \right) \right|;$$

In practice, these devices usually have two planet wheels arranged in parallel and displaced 180 degrees in phase, to help balance the system and minimize vibration. This practically means that the torques are shared between the two planet wheels, hence it has no relevant impact on the resulting Latent Power Ratio.

##
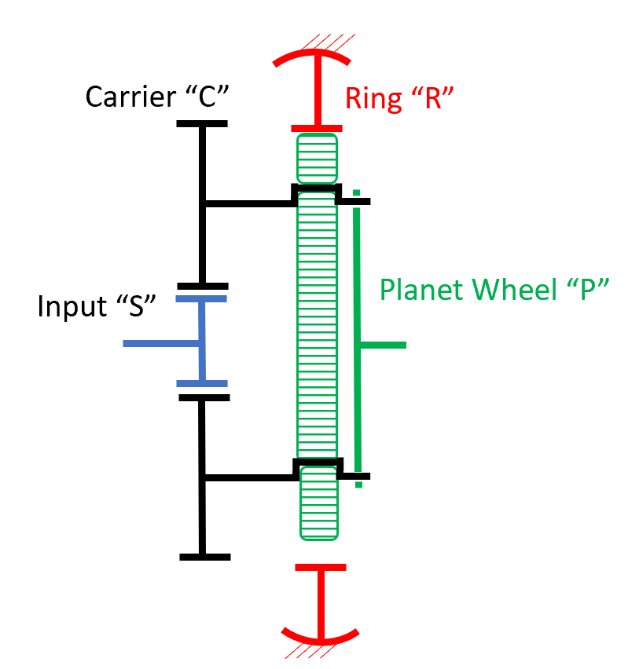
Cycloid Gearbox (CG) with Pre-Gearing

Introducing a pre-gearing stage into a conventional Cycloid, results in a more complex configuration which is best understood by assimilating the role of the planets of the pre-gearing to the carrier for the cycloid. The output shaft extracts the rotation of the planet-wheel, just like for the pure cycloid.

- Coupled: ${\omega"}_{c}=\omega_{c}= {\omega"}_{s}={\omega"}_{R};$
- Rolling: ${\omega'}_{c}=0$; ${\omega'}_{R}=\omega_{R}-{\omega"}_{R}=-\omega_{c}=\frac{Z_{P}}{Z_{R}}{\omega^{'}}_{P}=\frac{Z_{P}}{Z_{R}}\left( {\frac{1}{i_{CG}}\omega}_{S}-\omega_{c} \right); \omega_{c}=-\frac{Z_{P}}{\left( Z_{R}-Z_{P} \right)}\frac{1}{i_{CG}} \omega_{s};$

And the Latent Powers:

$${P^{'}}_{R-P}=-{P^{'}}_{R-P}=-\tau_{P}{\omega^{'}}_{P}=\tau_{S}i_{CG}\left( \omega_{P}-\omega_{c} \right)=P_{S}\left( \frac{Z_{R}}{Z_{R}-Z_{P}} \right);$$

$${P'}_{S-C}=\tau_{S}{\omega^{'}}_{S}=\tau_{S}\omega_{s}\left( 1+\frac{Z_{P}}{\left( Z_{R}-Z_{P} \right)}\frac{1}{i_{CG}} \right)=P_{S} \left( 1+\frac{Z_{P}}{\left( Z_{R}-Z_{P} \right)}\frac{1}{i_{CG}} \right);$$

And the Latent Power Ratio expressed as the sum of the absolute values of the Latent Powers on all meshings, divided by the Input Power results:

$$L_{CG}=\frac{\sum\left| {P'}_{j} \right|}{P_{S}}=\left( \frac{Z_{R}}{Z_{R}-Z_{P}} \right)+1+\frac{1}{i_{CG}}\frac{Z_{P}}{\left( Z_{R}-Z_{P} \right)}=\left( \frac{1}{Z_{R}-Z_{P}} \right)\left[ {2*Z}_{R}+Z_{P}\left( \frac{1}{i_{CG}}-1 \right) \right];$$

# Other approximations and criteria used in the assessment table

## Acceleration and Nominal torques

For the RG350 gearbox of ZF Friedrichshafen AG no data is available in terms of repeatable peak (acceleration) torques. Consequently, this value is extrapolated from the nominal value, using the average of the ratios between acceleration and nominal torques of the other two planetary gear train solutions.

## Latent Power Ratio

For the case of the Cycloid Drives RV-25N and Fine CYCLO F2C-T155, no precise data is available on the number of teeth of the planetary stage. The ratio between the number of teeth of the planets and the sun on that first stage is estimated to be 1:5, based on the gearbox topology and available figures.

## No Load Starting Torque

To estimate the no-load starting torques of the planetary gear trains a commonly used approximation for this type of gearboxes is applied, consisting on assuming that on the point of maximum efficiency, about 10% of the losses are load-independent, which is in good agreement with experimental results [Talbot and Kahraman, 2014], [Arigoni et al., 2010].
